# Drug Discovery Strategies for Kallikrein-Related Peptidases

**DOI:** 10.3390/ijms27010225

**Published:** 2025-12-25

**Authors:** Tobias Dreyer, Daniela Schuster, Viktor Magdolen, Peter Goettig

**Affiliations:** 1 Department of Gynecology and Obstetrics, Technical University of Munich, 81675 Munich, Germany; tobias.dreyer@tum.de (T.D.); viktor.magdolen@tum.de (V.M.); 2 Department of Pharmaceutical and Medicinal Chemistry, Institute of Pharmacy, Paracelsus Medical University, Strubergasse 21, 5020 Salzburg, Austria; daniela.schuster@pmu.ac.at; 3 Research and Innovation Center for Regenerative Medicine and Novel Therapies, Paracelsus Medical University, Strubergasse 21, 5020 Salzburg, Austria

**Keywords:** AlphaFold, cancer therapies, drug design, kallikrein, pharmaceuticals

## Abstract

Kallikrein-related peptidases (KLKs) are hallmarks of higher vertebrates, in particular of mammals. While the 15 human KLKs occur in nearly all tissues and body fluids and participate in many physiological processes, they are also involved in severe diseases. Among them are prostate, ovarian and breast cancer, as well as inherited skin and neurological disorders. Thus, KLKs have become targets for inhibitory compounds in academic and commercial research. The most prominent clinical biomarker and anti-cancer target for various approaches is PSA/KLK3. Already in the distant past, natural crude extracts were the source of medicine, while purified natural compounds and their derivatives are still the basis of about 50% of all pharmaceuticals. Nevertheless, structure-based rational design and high-throughput screening of natural and synthetic compound libraries are highly effective approaches for discovering lead compounds in the development of new drugs. Recently, computer-aided virtual or in silico screening has become a rapid method for such discoveries when combined with in vitro assays using protein targets or tests in cell cultures. To date, the successful implementation of artificial intelligence (AI) in the biosciences has significantly contributed to drug discovery. Our review focuses on state-of-the-art strategies and techniques in the context of KLK targets.

## 1. Introduction

About 330 million years ago, kallikrein proteases originated, as corroborated by a homolog of tissue kallikrein KLK1 found in amphibia, namely the frog *Xenopus tropicalis* [1]. In line with this evolutionary scheme, three reptilian and two avian KLKs were discovered. From the corresponding ancestor, kallikrein-related peptidases (KLKs) evolved into nine KLKs of marsupials and thirteen KLKs of all placental mammals. Most primates possess altogether 15 KLKs, whereby in humans they are encoded within a single gene cluster on chromosome 19 (locus 19q13.3–13.4). They are distantly related to plasma kallikrein (KLKB1), a trypsin-like serine protease, which regulates blood pressure similar to KLK1 [2]. The eponymous tissue kallikrein was discovered in 1926 by Emil Frey and Eugen Werle, while it was further investigated by Heinrich Kraut, who named it after the ancient Greek synonym for pancreas, καλλίκρεας [3]. Both KLKB1 and KLK1 regulate blood pressure by cleaving the bradykininogens high molecular weight kininogen (HMWK) and low molecular weight kininogen (LMWK); however, most KLKs have multiple physiological functions. According to the MEROPS database, the 15 trypsin- and chymotrypsin-like KLK proteases belong to family S1 and clan PA [4].

**Figure 1:**
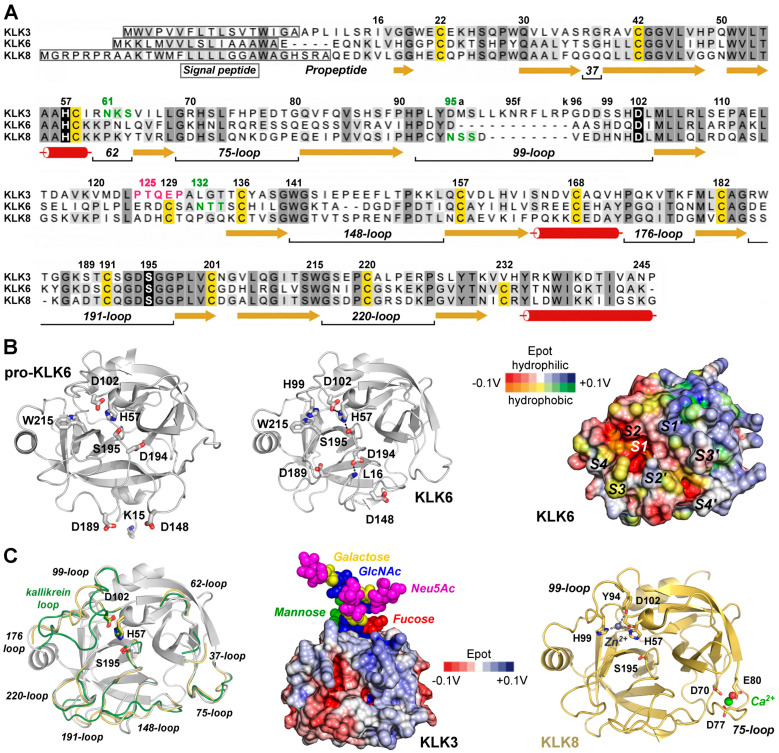
Exemplary KLK structures. (**A**) Sequence alignment of the pre-pro-KLKs 3, 6, and 8 labeled according to the chymotrypsinogen numbering. Darker background indicates high similarity, while the catalytic triad H57, D102, and S195 is shown with black background. α-helices (tubes), β-strands (arrows), and loops (lines) are displayed, with loops defined by their central residue, e.g., 37 or 75. The 99-loop of KLK3 with the insertion of 95a to 95k is known as *kallikrein loop*. Cys that form disulfides are highlighted with yellow background. Sequons with confirmed *N*-glycosylation are displayed in green and the *O*-glycosylation motif around T125 of KLK3 in magenta. (**B**) Comparison of pro-KLK6 (PDB code 1GVL, left panel) and KLK6 (1LO6, middle panel), whereby the specificity pockets on the molecular surface of KLK6 are indicated from S4 to S4′ (right panel). Polar and hydrophobic regions are depicted according to their electrostatic potential (Epot) from −0.1 V to +0.1 V in red, white (neutral), and blue or orange, yellow (neutral), and green, respectively. The surface Epot was calculated with the eF-surf server and visualized with the integrated Molmil software [28,29]. (**C**) Overlay of KLK6 (white) with surface loops of KLK3 (3QUM, green) and KLK8 (5MS3, orange). Middle panel: Molecular surface of KLK3 with polar Epot and the branched *N*-glycan linked to N61 represented as spheres for N-acetylglucosamine (GlcNAc), mannose, fucose, galactose, and N-acetylneuramidinic acid (Neu5Ac, magenta), a sialic acid. KLK8 is displayed with the stimulatory Ca^2+^ (green) bound to the 75-loop and the modeled inhibitory Zn^2+^ (gray) coordinated by H57, Y94, H99, and D102, thereby disrupting the catalytic triad. Tyr-Oη is an unusual ligand for Zn^2+^, but it was confirmed for the metallo-proteinase astacin [30]. Otherwise, Y94 could stabilize a water molecule as a fourth Zn^2+^ ligand.

Basically, KLK mRNAs and proteins are found in all tissues and fluids of the human body, with the notable exception of adult nerves according to a pioneering study and several databases (Table 1) [5,6]. All KLKs are expressed as pre-pro-proteases with a signal peptide that ensures threading of the nascent polypeptide into the endoplasmic reticulum, followed by *N*-glycosylation, folding, disulfide formation, quality control, transport to the Golgi system, *O*-glycosylation, and storage in secretory vesicles as zymogens (Figure 1A) [7]. Disulfides are formed between cysteines 22–157, 42–58, 129–232, 136–201, 168–182, and 191–220, whereby Cys129 and Cys232 are missing in KLKs 1, 2, 3, and 13. All pro-KLKs are secreted into the extracellular environment, often upon distinct hormonal signals [8]. To date, the only example of a true KLK zymogen determined by X-ray crystallography is human pro-KLK6, whereas a KLK/PSA structure (3QUM) adopts a zymogenic, inactive conformation induced by antibody binding [9,10]. Cleavage of the propeptides after Arg, Lys, or Gln (KLK4), and Phe (KLK13) in position 15 by proteases results in the activation of the KLK proteases, whereby a functional catalytic triad is formed consisting of His57, Asp102, and Ser195 according to the chymotrypsinogen numbering (Figure 1B) [11]. While the overall tertiary structure of the 15 mature KLKs is very similar to trypsin, chymotrypsin, or elastase, major differences are located in the eight surface loops surrounding the active site, which confer distinct specificity and elements of regulation (Figure 1A,C) [12]. Among these elements are glycosylated sequons and cation binding sites, as corroborated for a stimulatory Ca^2+^ and an inhibitory Zn^2+^ binding site in KLK8 (Figure 1A,C) [13]. The function of the majority of these glycans is not known, except for KLK2, with an *N*-glycan linked to Asn95 serving as regulator of substrate binding [14]. However, significant changes in the *N*-glycosylation patterns were observed for Asn61 of KLK3/PSA in prostate cancer, including an additional *N*-glycosylation site in the Asp95Asn variant, and for Asn132 of KLK6 in ovarian cancer (Figure 1C) [15,16,17]. These aberrations seem to have at least some diagnostic value, whereas the observed *O*-glycosylation of KLK3 at Thr125 and the *N*-glycan inked to Asn95 of KLK8 await further investigation [10,18].

Since all KLKs are involved in various diseases, in particular, many types of cancer, they have become preferred drug targets in medicinal and pharmaceutical research [19]. The best characterized pathophysiological roles of KLKs are found in prostate cancer (PCa), ovarian and breast cancer, as well as in skin diseases [19,20]. As natural KLK expression is already regulated on the epigenetic level by associated micro-RNAs, new pharmaceutical approaches are possible with therapeutic miRNAs and small interfering RNA (siRNA) [6,21]. Small nuclear polymorphisms of miRNA (miSNRPs) can serve as specific biomarkers and therapeutics for PCa in a personalized medicine [22]. In the following, we describe the current pharmaceutical strategies involving KLKs, with a focus on inhibitory molecules. Overviews of KLK natural and synthetic inhibitors are available from various research groups [7,23,24].

**Table 1:** Selected tissue or body fluid expression of kallikrein-related peptidases (KLKs) according to three relevant sources. Comparisons between KLKs are difficult, e.g., as the concentration of KLK3/PSA in seminal plasma is about 100-fold higher than the one of KLK2. * mRNA data.

KLK	mRNA (Gene Atlas) [25,26]	Protein [5]	Human Protein Atlas [27]
KLK1	pancreas, salivary gland, kidney	pancreas, salivary gland	pancreas, intestines, salivary gland
KLK2	prostate	prostate, seminal plasma	prostate
KLK3	prostate	prostate, seminal plasma	prostate
KLK4	prostate	semen, cervix, pituitary, muscle	prostate
KLK5	skin, testis, heart, liver, tongue	skin	skin, testis
KLK6	brain, spinal cord	brain	brain
KLK7	skin, liver, tongue	skin, liver, heart, esophagus	skin, bone marrow
KLK8	skin, tongue	skin, tonsil, esophagus, breast	skin, oral mucosa, vagina, brain
KLK9	adrenal cortex	heart	skin, oral mucosa, vagina
KLK10	skin, tongue	skin, tonsil, esophagus	esophagus, vagina, cervix, skin *
KLK11	prostate, trachea, tongue, skin	prostate	skin, esophagus, salivary gland
KLK12	tongue	bone, intestines, vagina, lung	esophagus, vagina, cervix *
KLK13	kidney, tongue	tonsil, esophagus, vagina, cervix	skin, esophagus
KLK14	skin	skin, vagina, breast, bladder	skin, vagina, cervix, brain *
KLK15	uterus, liver	skin, liver, kidney, heart, breast	salivary gland, intestines, testis *

## 2. General Strategies of Drug Discovery

### 2.1. A History from Ancient Natural Drugs to Modern Pharmacology in a Nutshell

The primeval origins of pharmacology and medicine are demonstrated by the remarkable example of chimpanzees, who apply plant leaves as self-medication [31]. Prehistoric humans used plants and fungi as remedies, like the 5000-year-old Tyrolean Iceman [32]. Ancient Egyptian medicine utilized a wide array of plant-based remedies in diverse forms, demonstrating a sophisticated understanding of pharmacology [33]. For example, willow bark containing salicin was used to treat inflammation and pain. Greeks and Romans continued the Egyptian medicine and pharmacology by exploiting natural resources [34]. Similarly, medicine and pharmacology developed in many cultures, in particular, in Indian Ayurveda and traditional Chinese medicine [35,36]. Significant progress was made by the Arabs in their Golden Age from 750 to 1258 CE [37]. Scholars of the Islamic world wrote a vast medical and pharmacological literature and established a system of pharmacies, while Medieval Europeans mixed corresponding Greek, Roman, and Islamic advancements [38].

The age of enlightenment paved the way for the natural sciences and modern pharmacology [39]. Since the 19th century, natural compounds have been isolated by means of chemistry and the concept of their bioactivity has emerged. Also, the first pharmaceuticals were synthesized, such as the hypnotic chloral hydrate and the pain killer aspirin, being acetylated salicylic acid [40,41]. The foundations for the screening of libraries with natural and synthetic compounds were laid in the late 19th century [42]. Chemical compounds that targeted a specific pathogen without side-effects were called *Zauberkugel* or “magic bullet” by Paul Ehrlich [43]. Thus, the first successful chemotherapy was developed in 1909, using the arsenic organic compound Salvarsan to treat syphilis.

### 2.2. From Low-Throughput to High-Throughput Screening of Compound Libraries

Until the 1940s, efficient drugs were discovered by screening natural or synthetic compounds at random [44]. Modern organic synthesis revolutionized drug discovery in the 1950s, which was facilitated by the development of the quantitative structure-activity relationship (QSAR) in the 1960s [45]. An overview including many methods of pharmacology focuses on the highly relevant ligand–receptor concept [46]. From 1990 onwards, high-throughput screening (HTS) of natural, synthetic, and mixed libraries became the most efficient method [47]. Driven by the pharmaceutical industry, both automation and combinatorial organic chemistry with solid phase or solution syntheses enormously accelerated HTS [48]. A computational descendant of the HTS method is the virtual or in silico screening of libraries with millions of compounds. 

### 2.3. Rational Design of Synthetic Inhibitors as Lead Compounds

The so-called rational design method is based on experimental structures and substrate analogs as inhibitors [49,50]. In order to find a novel lead compound, previously solved target–ligand complex structures are very helpful. Starting with this specific inhibitor, a small set of derivatives is synthesized or investigated from purified related natural molecules. Fragment-based drug discovery (FBDD) is mostly based on high-resolution X-ray crystal structures, comprising fragments of protein binding groups, aiming at generating nanomolar lead compounds [51]. Hereby, the concept of “sociability” identifies fragments with many connections in the drug discovery context, whereas “unsociable” fragments, which are rarely mentioned, could be valuable new building blocks of drugs. In addition, fragment elaboration might discover cryptic sub-pockets that form upon binding of ligands or regulatory biomolecules. A more recent variant of rational design is the de novo design, which is based on experimental structures and tries to calculate an optimal binding inhibitor since the early 1990s [52].

### 2.4. In Silico Screening of Compound Databases

Recently, a general overview of computer-aided drug design (CAAD) explained the most essential requirements of state-of-the-art methods based on advanced computing [53]. Computational approaches allow for the so-called in silico or virtual screening of millions of molecules from various databases that contain commercially available compounds [54]. One of the most important ones is the ZINC database, which comprises holarctisover 230 million purchasable compounds in ready-to-dock 3D formats [55]. Independent from the in silico method used for screening, the theoretical workflows need to be developed and validated using already known active and inactive compound data. In the last decade, large bioactivity databases emerged that can serve as resources for training data sets. Among these databases are the BindingDB, which contains 1.3 million compounds with measured binding affinities, the ChEMBL database with 2.4 million manually curated molecules, and PubChem with around 300 million substances including their bioactivities [56,57,58].

For virtual screening, structure- and ligand-based approaches are available. Structure-based approaches such as molecular docking or molecular dynamics (MD) simulations use experimental or calculated 3D structures of the target and estimate a compound’s fitting or binding behavior. Ligand-based methods such as pharmacophore modeling or shape-based screening compare the properties of active ligands to those of the screening compounds, searching for common chemical functionalities, sizes, shapes, and also physicochemical properties. Compounds fitting into the binding site and/or sharing properties with active compounds have a higher probability to show activity in a respective in vitro experiment compared to randomly selected compounds [59].

Virtual screening and property prediction tools are often combined for improving their performance and making the workflow computationally more efficient. For example, three-dimensional (3D) pharmacophore models of bioactive compounds are generated with LigandScout, which uses data from protein-bound ligands and can be applied to experimental structures of the PDB or to homology models [60]. Then, the Genetic Optimization for Ligand Docking software (GOLD) allows for ligand docking with the full range of its conformational flexibility and with partial flexibility of the protein, as well as considering the displaced water molecules from the binding sites [61]. Selected hits can be filtered with the free SwissADME server with respect to pharmacokinetics, i.e., absorption, distribution, metabolism, and excretion in organisms [62]. More recently, artificial-intelligence-based methods have been implemented in both structure-based and ligand-based approaches [63].

**Figure 2:**
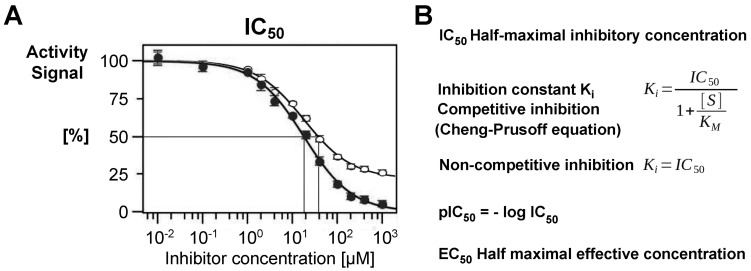
(**A**) The IC_50_ value is one of the central parameters in inhibition kinetics. It is defined as the half-maximal inhibitory concentration, i.e., the concentration of a compound that reduces the activity of an enzyme to 50%. In the displayed scheme, the inhibitor (●) has an IC_50_ of about 20 µM. If a residual activity is observed (○), the relative IC_50_ can still be 20 µM, but the absolute IC_50_ is significantly higher. (**B**) In competitive inhibition, the K_i_ is a more reliable parameter, since it takes into account the substrate concentration [S] and the Michaelis constant K_m_, which is [S] at the half-maximal rate constant of an enzymatic reaction. The pIC_50_ is a pure number derived from the IC_50_. By contrast, in non-competitive inhibition, the K_i_ corresponds exactly to the IC_50_. A more general parameter is half-maximal effective concentration (EC_50_) values that can be used for any dose–response/effect as a measure for potency in pharmacology, e.g., for stimulating compounds.

This general approach works on the 2D and 3D level on various scales, such as for computational screening of about 9000 antiviral compounds which yielded four hits with favorable ADME parameters binding to the SARS-CoV-2 main protease (Mpro) [64]. A general, more “rational” process for in silico screening using prior knowledge of inhibitory parameters and target coordinates from the PDB is described by Horgan and coworkers, who characterized a tubulin inhibitor and potential lead with an in vitro IC_50_ of 2.9 µM (Figure 2) [65]. Probably, combinations of the above-described approaches can be even more successful, as recently described in a comparative study to find new antibacterial compounds [66]. A high-throughput screening of about 2 million small molecules directed against an *E. coli* strain resulted in thousands of hits, which were used to train the deep learning model GNEprop to predict antibacterial activity. Subsequently, in silico screening of 1.4 billion synthetically available compounds eventually yielded 82 compounds with significant antibacterial activity. All the previously described strategies to find bioactive compounds require in vitro and in vivo assays, such as in cell cultures. Ideally, structural studies with X-ray crystallography, NMR, and cryo-EM confirm the functional data and can provide information that allows for optimizing lead compounds or established drugs. In the following, we describe three major classes of inhibitory biomolecules beyond peptidic and other small molecules.

### 2.5. Natural Proteinaceous and Polypeptidic Inhibitor Scaffolds

Natural proteinaceous inhibitors belong to the category of biologics, for example, antibodies, but not nucleic-acid-based aptamers. Several therapeutic approaches can be mentioned, which were approved by the Food and Drug Administration (FDA) in the US or by the European Medicines Agency (EMA). Already in 1987, the FDA allowed α-1-antitrypsin (AAT, MEROPS ID I04.001) for the treatment of emphysema associated with AAT deficiency [67]. Aprotinin or bovine pancreatic trypsin inhibitor (BPTI, I02.001) was widely applied under the name Trasylol by transfusion for many types of surgery to prevent blood loss since its approval by the FDA in 1993, but its usage has been limited due to side effects [68]. By contrast, the direct thrombin inhibitors lepidurin, bilavirudin, and desidurin prevent blood clotting like their parent substance hirudin from the leech *Hirudo medicinalis* even in the femtomolar range [69]. They are mostly used in non-surgical procedures with catheters or to prevent and treat deep vein thrombosis. Regarding the KLKs, many studies have tried to engineer natural inhibitors in academic research aiming at pharmaceutical applications. For example, based on the Kunitz inhibitor domain from the human amyloid β-protein precursor, engineered variants were directed at trypsin-3 (mesotrypsin) and KLK6 [70]. The inhibitor mutants APPI-3M and APPI-4M were efficient in preclinical models of PCa and breast and ovarian cancer, respectively, whereby they accumulated in tumors with long retention and acceptable pharmacokinetics. The sunflower trypsin inhibitor (SFTI-1) is a frequently used 14-residue cyclic peptide, with many applications in research and the potential to develop inhibitors as therapeutics, e.g., directed against the Mpro cysteine protease of the coronavirus SARS-CoV-2, which exhibits a chymotrypsin fold [71]. Due to the thermal resistance and long half-life of wt-SFTI-1 in human serum, it appears to be predestined as lead scaffold for engineered drugs [72].

### 2.6. Antibody-Based Inhibitors

**Figure 3:**
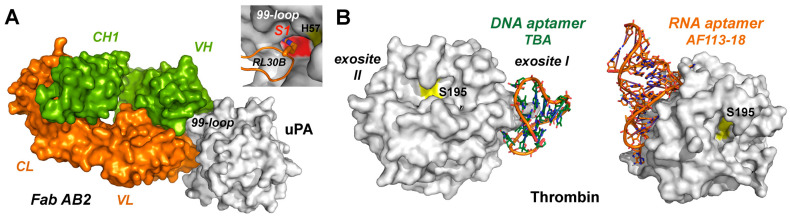
(**A**) The inhibitory Fab-AB2 antibody binds with an Arg (RL30B) of the L1 loop in the variable light chain (VL) to the S1 pocket of the trypsin-like protease uPA (PDB 9PYF). An IC_50_ of 8 nM was reported, whereby the variable region of the heavy chain (VH) binds to the 99 and 175-loops, and the constant regions CL and CH1 are labeled as well. The Fab molecular surface of the light chain is shown in orange and the one of the heavy chain in green. (**B**) Aptamers as inhibitors of the blood coagulation factor thrombin, whose catalytic Ser195 is depicted as a yellow patch. The 15-nt DNA aptamer TBA in complex with thrombin essentially binds exosite I, which is positively charged as exosite II (4DIH). The exosite II binding 25-nt RNA aptamer AF113-18 was engineered with a phosphorodithioate that conferred high inhibitory potency corroborated by a K_D_ of 1.8 pM (5DO4).

Also, antibodies are considered as drugs nowadays; however, clinical trials and therapies usually do not employ inhibitory antibodies but rather more elaborate immunological and medical approaches, which are approved by the FDA or EMA. Due to the relatively easy engineering and purification process, mostly fragment-antigen-binding variants (Fab) are used, which are derived from IgGs. For the engineered Fab variant U33-AB2, which inhibits the trypsin-like urokinase plasminogen activator (uPA), an IC_50_ of 8 nM and a K_D_ of 5 nM were reported (Figure 3A) [73]. The dissociation constant K_D_ largely corresponds to the inhibition constant K_i_ and was used to evaluate the potency in a series of Ala and Arg scanning of Fab variants in the six complementarity-determining regions (CDRs). Unequivocally, an Arg residue in the L1 loop of the light chain fully occupies the S1 subsite in a substrate-like manner, while similar binding modes were observed for inhibitory antibodies with the trypsin-like serine proteases HGFA-1, MT-SP1, and blood coagulation factor IX.

An example of KLK targeting monoclonal antibodies (mFab) is DX-2300, which inhibits KLK1 in the lower picomolar range [74]. Thus, inhibitory antibodies could become highly specific alternatives to small molecule inhibitors. For example, a bispecific anti-KLK5/7 Fab antibody was raised against murine KLK5 and KLK7 with the potential to treat Netherton syndrome (NS) [75]. In addition, camelid single-domain antibodies or nanobodies consisting only of the variable IgG heavy chain domain can be very effective active site inhibitors, as corroborated by the urokinase plasminogen activator (uPA) Nb4 pair [76]. It exhibited a K_i_ of 1.2 nM and showed fine details of Nb4 binding with the P1-Arg inserted into the S1 pocket of the trypsin-like serine protease uPA, similar to bovine pancreatic trypsin inhibitor (BPTI). Monoclonal antibodies directed at the less conserved 75-loop epitope and full-length KLK4 had EC_50_ values near 270 pM for the degradation of fibrinogen and significant inhibitory effects on proliferation and migration of ovarian carcinoma cells [77].

### 2.7. DNA and RNA Aptamers

Aptamers are engineered single- or double-stranded DNA, RNA, XNA, or peptide molecules, which are chemically synthesized and optimized by the “systematic evolution of ligands by exponential enrichment” (SELEX) or related processes [78,79]. Overall, in the wider context of oligonucleotides, various clinical and therapeutic strategies exist based on small interfering RNA (siRNA) antisense oligonucleotides (ASOs), short activating RNA (saRNA), as well as DNA and RNA aptamers [80]. However, only two ophthalmological therapies using aptamers are currently approved by the FDA. Since 1992, when the first thrombin targeting DNA aptamer was found to inhibit the procoagulant activity of thrombin, many applications of this approach have been found for anticoagulation and homeostasis [81]. Several DNA and RNA aptamer–thrombin complexes were structurally determined, with IC_50_ values in the picomolar range, binding either exosites I or II, as the 15-nt TBA-aptamer, which exhibits an unusual DNA quadruplex stacking (Figure 3B). Binding constants are well in the low nanomolar or even picomolar range, as corroborated by the 25-nt RNA aptamer AF113-18, with a K_D_ of 1.8 pM for α-thrombin [82]. Although DNA and RNA aptamers targeting KLK3/PSA and KLK6 were already generated from 2010 onwards, not much progress was made with clinical trials [6].

Nevertheless, such aptamers are intensively studied by many academic groups with respect to diagnostic, prognostic, and theranostic research in the context of PCa, often involving PSA-containing samples [83]. Theranostics can be defined as largely personalized medicine with radionuclide imaging and radiation therapy. In line with this development, aptamer-based biosensors, which employ new nanomaterials, dual-modal detection, and CRISPR-Dx technology, have improved the sensitivity and accuracy of PSA detection in patient samples. Thus, personalized diagnosis and therapies can certainly further improve in the near future [84]. Recently, in silico screening and optimization of PSA binding DNA aptamers and their kinetic analyses were performed, which could contribute to faster and better SELEX processes [85].

## 3. Specific KLK Physiology and Their Relevance as Therapeutic Targets of Inhibitors

**Figure A1:**
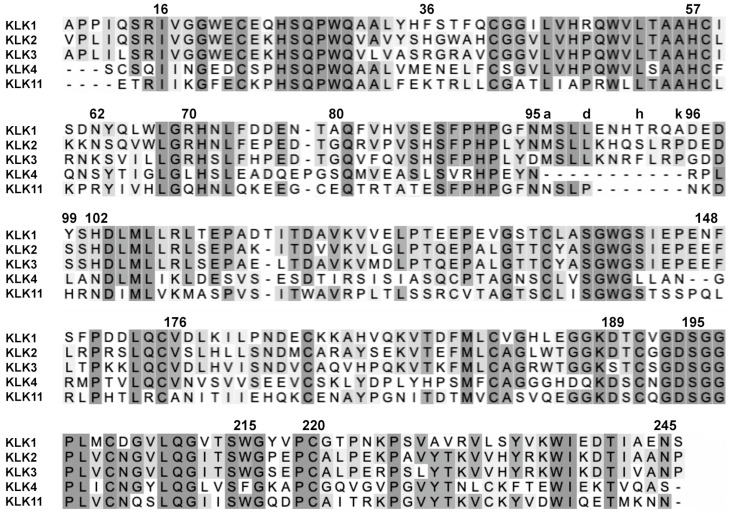
Sequence alignment of pro-KLK1 and prostatic pro-KLKs with *Clustal Omega.* Higher similarity is represented by darker shades of gray. The terminal residues 16 and 245 of the mature proteases are marked, as well as the catalytic triad (57, 102, 195). In addition, residues defining important loops are labeled (70–80, 99, and 148) and the residue 215 at the bottom of the S4 pocket.

**Figure A2:**
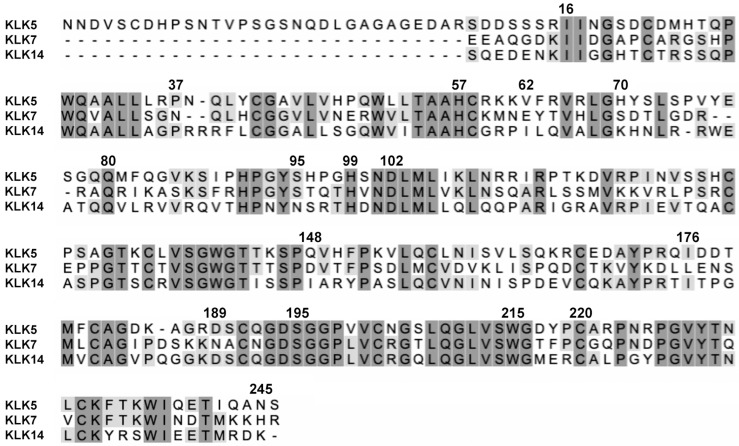
Sequence alignment of skin-derived pro-KLKs with *Clustal Omega*. Higher similarity is represented by darker shades of gray. The terminal residues 16 and 245 of the mature proteases are marked, as well as the catalytic triad (57, 102, 195). In addition, residues defining or near important loops are labeled (37, 62, 70–80, 99, 148, 176, 220) and the residue 215 at the bottom of the S4 pocket.

**Figure A3:**
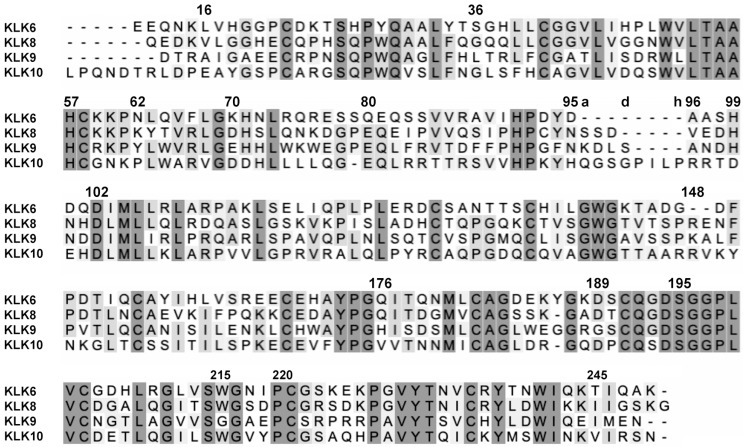
Sequence alignment of brain- and neuron-derived pro-KLKs with *Clustal Omega*. Higher similarity is represented by darker shades of gray. The terminal residues 16 and 245 of the mature proteases are marked, as well as the catalytic triad (57, 102, 195) and the P1-specificity determining D/G189. In addition, residues defining important loops are labeled (99, 148, 176, 220) and the residue 215 at the bottom of the S4 pocket.

**Figure A4:**
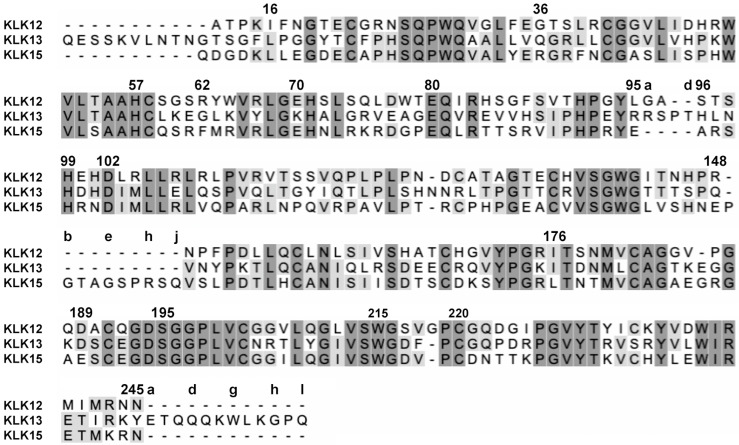
Sequence alignment with *Clustal Omega* of pro-KLKs without clear allocation to a group. Higher similarity is represented by darker shades of gray. The terminal residues 16 and 245 of the mature proteases are marked, as well as the catalytic triad (57, 102, 195) and the P1-specificity determining D/E189. In addition, residues defining important loops are labeled (99, 148, 176, 220) and the residue 215 at the bottom of the S4 pocket. KLK13 and KLK15 exhibit a unique 12 residue extension of the C-terminus and a 10-residue insertion of the 148-loop, respectively.

At least one major physiological function of most KLKs is well understood, while many of them have multiple tasks, corresponding to their distribution in tissues and body fluids (Table 1). Four sequence alignments of functional KLK groups are displayed in Appendix A, comprising KLK1 and the prostatic KLKs 2, 3, 4, and 11 (Figure A1), the skin-derived KLKs 5, 7, and 14 (Figure A2), the brain-related KLKs 6, 8, 9, and 10 (Figure A3), as well as the more isolated KLKs 12, 13, and 15 (Figure A4). Table 2 lists both information on established or very likely physiological functions, as well as non-cancer diseases and disorders. Essentially, the most important pharmaceutical approaches with respect to KLKs and their involvement in cancer and other diseases were reviewed by Sotiropoulou and Pampalakis in 2012, while the group of El-Amri wrote an overview with the focus on KLK inhibition in 2018 [24,86]. Furthermore, a variety of activity-based probes has been developed, which mostly serve for imaging and other purposes, while they often possess inhibitory properties [87,88]. Focusing on KLKs as therapeutic targets, Daneva and colleagues recently presented a comprehensive review, which also gives a good general introduction to all aspects of human KLKs [19].

Basically, all KLKs are involved in cancer processes, whereby KLK12 mRNA transcripts were detected in many cancers, but not as protein (Table 3) [27]. However, recently, KLK12 was detected at significant concentrations in breast cancer tissue by immunostaining [116]. It is noteworthy that KLK14 is found in the cytoplasm of nearly all cancers.

### 3.1. Tissue Kallikrein KLK1 in Blood Pressure Regulation and Lung Diseases

Tissue kallikrein KLK1, the eponymous serine protease of the kallikrein-related peptidase family encompassing fifteen secreted members (KLK1-15), is a central enzymatic component of the kallikrein–kinin system (KKS). By cleaving mainly low-molecular-weight kininogen to release bradykinin, KLK1 promotes vasodilation, whereby an imbalance of the KKS and the renin–angiotensin system (RAS) causes hypertension, in addition to improving microcirculatory perfusion and modulating inflammation [117]. This mechanism is vital for vascular homeostasis and blood pressure regulation. Deficiency or dysregulation of KLK1 alone can result in hypertension and is linked to atherosclerosis, as well as microvascular complications in diabetes [118,119]. High KLK1 levels represent a strong predictor for the severity of coronary artery disease (CAD). In addition, KLK1 is involved in asthma pathogenesis with KLK3 and KLK14 [90].

A study with mouse models of ischemic tissue corroborated that KLK1, together with MMP-9, is important for vascular repair and neovascularization [120]. In the cardiovascular context, kinins released by KLKB1 and KLK1 ensure a regulated papillary blood flow with excretion of water and sodium, while KLK1 acts as a protective vasoregulatory factor, balancing vascular tone and limiting ischemic injury [121]. Preclinical and clinical studies suggest that insufficient KLK1 activity can exacerbate vascular stiffness, impair endothelium-dependent relaxation, and increase stroke risk. Conversely, adenoviral vectors for KLK1 gene incorporation and further protein infusion into tissues of animal models could even reverse myocardial, renal, and cerebrovascular ischemic tissue damage [122]. Intravenous or subcutaneous administration of KLK1 itself as recombinant protease, termed DM199, showed promising results regarding the safety of future treatment of acute ischemic stroke (AIS) [123]. Similarly, therapeutic KLK1 supplementation or enhancement of its activity has shown promise in restoring endothelial function and lowering blood pressure in experimental models. Recently, both natural human urinary kallikrein (HUK), misnamed kallidinogenase in some Chinese publications, and recombinant KLK1 were successfully applied in treating AIS [124,125].

Beyond the cardiovascular system, KLK1 plays a pivotal role in pulmonary physiology [90]. In the airways, elevated KLK1 activity has been detected in asthma patients, where it contributes to goblet cell metaplasia and mucus hypersecretion, hallmarks of airway obstruction. Activation of KLK1 in the lung can be triggered by degradation of hyaluronan, a matrix component that sequesters the inactive enzyme. Once released, active KLK1 may stimulate EGF signaling and increase oxidative stress susceptibility, processes implicated in chronic obstructive pulmonary disease (COPD) progression. Genetic studies highlight a nuanced role for KLK1 in lung disease: certain polymorphisms appear to reduce the risk of onset of COPD, while lower KLK1 levels in established COPD may worsen outcomes upon viral infections [126]. Notably, KLK1 can inhibit influenza viral entry by cleaving hemagglutinin and enhance innate immune responses via macrophage and NK cell activation [9]. These findings position KLK1 as both a pathogenic factor in chronic airway inflammation and a protective factor in pulmonary immunity [127,128]. Moreover, patients with severe symptoms of COVID-19, such as pulmonary edema and thromboembolic complications, exhibited elevated KLKB1, KLK1, and kinin levels in bronchoalveolar lavage fluid and corresponding activity [129]. This dysregulation of the kallikrein–kinin system is caused by binding of the viral spike protein of the SARS-CoV-2 to the angiotensin-converting enzyme 2 (ACE2) on alveolar epithelial and vascular endothelial cells and further signaling. Thus, inhibition of the kallikrein–kinin system could be a potential treatment for severe COVID-19. Based on several related studies, therapeutic approaches were proposed for the targets KLK1, KLKB1, coagulation factor XII, bradykinin, bradykinin B1 and B2 receptors, as well as others [91].

**Figure 4:**
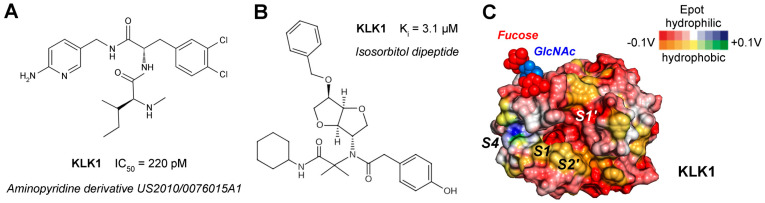
(**A**) Aminopyridine inhibitor of KLK1 from a US patent with a picomolar IC_50_. (**B**) An isosorbitol derivative that was obtained by an Ugi multicomponent reaction, which occupies the S2, S1′, and S2′ pockets in MD simulations, but not S1. (**C**) Molecular surface of KLK1 (PDB code 8YGY) with electrostatic potential (Epot from the eF-surf server) calculated from −0.1 to +0.1 V, whereby red and blue define polar regions and orange to green hydrophobic ones, respectively. In line with its acidic pI of 4.6, the surface of KLK1 is predominantly negatively charged, except for the positively charged S4 pocket. The *N*-glycan linked to Asn95 may regulate substrate access to the active site as in KLK2 [14].

KLK1 is also emerging as a risk gene for the rare pulmonary arterial hypertension (PAH) with high mortality, though causal mechanisms remain under study [130]. In inflammation and vascular remodeling processes, KLK1 activity is naturally regulated by kallistatin, which is encoded by the *SERPINA4* gene. Recombinant kallistatin exhibited a k_app_ of 2.6 × 10^3^ M^−1^ s^−1^; however, binding of kallistatin to heparin may reduce its protective potential as suggested by their complex crystal structure [131]. One of the strongest known synthetic inhibitors is an aminopyridine derivative exhibiting two peptide bonds with high selectivity over KLKB1 and a K_i_ of 220 pM, described in the patent US2010/0076015A1 (Figure 4A) [86,132]. Regarding small molecule inhibitors agreeing with the “rule of 5”, peptidomimetic isomannide derivatives were synthesized de novo, with an aminopyridine (10) exhibiting a K_i_ = 3.1 µM (Figure 4B) [133,134]. Docking and MD favor the binding of this compound from the S2 to the S2′ subsites. According to predictions and assessments by the ADMET software modules, this molecule possesses a good bioavailability and little toxicity and, thus, may serve as a lead compound. The dual P1-preference for Arg and Tyr can be further exploited, as well as the unique positively charged S4 pocket, which favors P4-Glu, as demonstrated in a phage display study (Figure 4C) [135]. A defarsirox (DFX) derivative, with 1,2,4-triazole core comprising phenolic groups and a benzyl group, inhibited KLK1 with a K_i_ of 24 µM, whose potency was significantly enhanced by chelating Fe^3+^ [136]. Also, the inhibitory monoclonal antibody DX-2300 targets KLK1 to treat diseases of the human airways, like asthma [74]. DX-2300 inhibits KLK1 in a competitive manner with a K_i_ = 130 pM. In addition, this antibody is active site binding and very specific as it does not inhibit 11 human KLKs and ten other crucial human serine proteases.

In summary, KLK1 serves as a bimodal regulator, essential for maintaining blood pressure stability and significantly influencing lung disease pathophysiology. Its enzymatic balance is critical: too little activity may impair vascular and pulmonary defense, while excessive activity can promote airway pathology. These dual roles make KLK1 an attractive target for precision therapies in cardiovascular and respiratory disorders.

### 3.2. Prostatic Kallikrein-Related Peptidases KLK2, KLK3, KLK4, and KLK11

The prostatic subgroup of the KLK family could be limited to KLK2, KLK3 (prostate-specific antigen, PSA), KLK4, and KLK11, whereby especially KLK2 and KLK3 are highly expressed in the prostate and secreted to seminal fluid [5]. Since prostate cancer (PCa) is the second most common cancer in men with 1.4 million new cases and 400,000 deaths every year, predominantly KLK3/PSA and to a lesser extent KLK2 have become diagnostic tools and drug targets [137,138]. The biomarker test using the concentration of PSA in serum for tracking PCA was introduced in 1986 and was the basis for FDA-approved follow-up tests [139]. PSA levels alone cannot discriminate between aggressive PCa, benign prostate hyperplasia (BPH), and inflammation of the prostate. By contrast, the Prostate Health Index (PHI), which measures total PSA, free PSA, and pro-PSA with a deletion (p2PSA), enhanced the diagnostic specificity and accuracy resulting in less unnecessary biopsies and surgery. Recently, the 4K-test further improved the efficacy and diagnostic performance for PCa by combining levels of total PSA, free PSA, intact PSA, and KLK2 [140]. However, other KLKs play a role in the prostate, such as KLK1, KLK5, KLK9, and KLK15, which are upregulated in prostate cancer [113,141]. Moreover, KLK5 is expressed in skin, testis, and at low levels in prostate and seminal plasma. The physiological functions of the prostatic KLKs center on the regulation of seminal plasma homeostasis, liquefaction of the seminal coagulum, and modulation of proteolytic cascades that maintain sperm motility [142]. The major substrate of KLK2 is the zymogen form of KLK3/pro-PSA, whereby cleavage by KLK2 results in its activation [143]. Active KLK3 hydrolyzes the semenogelins 1 and 2 together with KLK2, while KLK4 supports extracellular proteolysis of matrix proteins [144,145]. Due to the occasionally low concentration of KLK4 in the ng/mL range in seminal plasma, it is most likely another activator within the semen liquefaction cascade, with KLK2 and KLK3 reaching concentrations of 10 to 1000 µg/mL [93]. By contrast, both mRNA and protein levels of KLK4 were reported to be very high in prostate [6]. KLK5 and KLK11 may contribute to the broader protease networks in semen and may have roles beyond reproduction [146]. Regulation occurs at multiple levels, including hormonal control by androgens, estrogens, and progestins on the transcriptional level, as well as post-transcriptional control by miRNAs and modulation of enzymatic activity by metal ions such as Zn^2+^ or by endogenous protease inhibitors, notably serpins such as kallistatin [110,147,148]. Interestingly, eicosapentaenoic acid, an omega-3 fatty acid, induces miRNA-378, which exerts anti-PCa effects by targeting KLKs 2, 4, 6, and 14 [149].

In prostate cancer (PCa), these KLKs become dysregulated, contributing to tumorigenesis, progression, and therapy resistance. KLK2 and KLK3/PSA participate in extracellular matrix degradation, activation of protease-activated receptors, in particular of PAR-2, tumor invasion, angiogenesis, and proliferation [138,150]. As KLK3/PSA and KLK4 promote the epithelial–mesenchymal transition (EMT) of prostate cancer cells and their dissemination, KLK4 is also strongly linked to aggressive PCa, whereas knockdown of the *KLK4* gene significantly reduces PCa cell proliferation [151,152]. Furthermore, KLK4 increases angiogenic activity in PCa, while it activates MMP-1 and thrombospondin-1, which facilitates and promotes lethal PCa bone metastasis [145,153]. Across the board, KLK expression patterns correlate with tumor grade, Gleason score, serum PSA levels, and clinical outcomes. While several KLKs are subject to hormonal control, KLK15 is distinguished by its androgen-regulated expression, indicating a likely role in prostate physiology and disease as PCA [25,154].

**Figure 5:**
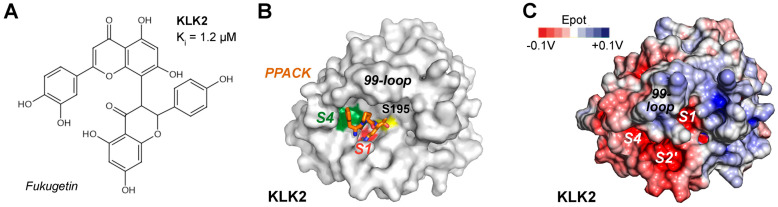
(**A**) The plant flavonoid fukugetin is an inhibitor of KLK1 and KLK2 in the low micromolar range. (**B**) Molecular surface of KLK2 (PDB 4NFF) in the open E conformation with colored S4 pockets near Trp215 (green) and S1 Asp189 (red). The inhibitor D-Phe-Pro-Arg-chloromethyl ketone (PPACK) is represented by sticks (orange), with P2-Pro occupying part of the S2 pocket. The catalytic Ser195 is represented by a yellow patch. (**C**) Molecular surface of the AlphaFold model for KLK2 with the defined 99-loop in a closed E* conformation. The electrostatic potential ranges from −0.1 V (red) to + 0.1 V (blue), whereby neutral zones are white.

KLK2 (S01.161) strongly favors P1-Arg over P1-Lys substrates, while the other subsites from S4 to S4′ are not very specific [14]. Synthetic cyclic 14-mer peptides, derived from the sunflower trypsin inhibitor (SFTI), significantly inhibited KLK2, as well as linear peptides such as ARFKVWWAAG with a K_i_ of 280 nM for insulin-like growth factor binding protein-3 (IGFBP-3) as substrate [155,156]. Fukugetin is a plant flavonoid from *Garcinia brasiliensis*, which exhibits a K_i_ = 1.2 µM for KLK2 and a slightly higher one for KLK1, while it may bind to the S4 to S1 specificity pockets (Figure 5A) [157]. These pockets are occupied by the chloromethyl ketone inhibitor PPACK, comprising a *D*-Phe, a Pro, and an Arg side-chain in the open E conformation of a KLK2 crystal structure with a flexible and disordered kallikrein 99-loop (Figure 5B) [158]. By contrast, the AlphaFold model shows the defined 99-loop in a closed E* conformation (Figure 5C). The model of conformational selection for trypsin-like serine proteases was developed by the group of di Cera, but has a general significance for the binding of substrates and inhibitors to enzymes [159].

A virtual screening study of nearly 12,000 phytochemicals identified the compounds phaseolin, physalin D, and nicandrenone as potential KLK2 inhibitors with favorable predictions for pharmacokinetics, no toxicity, and acceptable bioactivity [160]. The calculated dissociation constants K_d_ are around 30 µM, whereby molecular docking showed no specific binding to the S1 pocket, which may allow further modifications as lead compounds.

**Figure 6:**
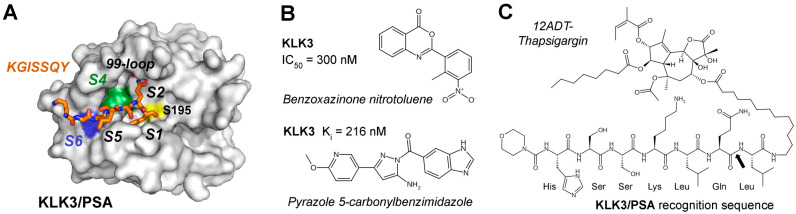
(**A**) Molecular representation of KLK3/PSA (PDB 2ZCK) in complex with an acyl intermediate of a cleaved substrate derived from semenogelin depicted as stick model. This peptide binds the S1 to S7 pockets, which are hydrophobic (green), positively charged (blue), or mixed polar and hydrophobic (white), with the catalytic S195 shown as yellow patch. Parts of the kallikrein/99-loop covering the active site are omitted. (**B**) Two competitive inhibitors of KLK3/PSA with nanomolar IC_50_ or K_i_ could serve as lead compounds for new anti-PCa drugs. (**C**) A most promising field of therapeutics is prodrug design for PSA. Cleavage at the recognition sequence of HSSKLQ↓L-12ADT releases the cytotoxic construct Leu-12ADT-thapsigargin. The scissile bond is indicated by a black arrow.

In contrast to KLK2, PSA (S01.162) is a chymotryptic peptidase due to its Ser189 at the bottom of the S1 pocket, which prefers P1-Gln, Tyr, and Leu residues, while the specificity in the non-prime side was corroborated by a substrate complex structure (2ZCK, Figure 6A) [161]. A high-throughput screening (HTS) study of 50,000 commercial compounds identified a benzoxazinone as KLK3/PSA inhibitor with an IC_50_ of 300 nM, as well as equally potent triazoles (Figure 6B) [162]. Similarly, many peptide β-lactam, boronate, and aldehyde inhibitors mostly based on semenogelin substrates were investigated, exhibiting K_i_ values for PSA in the low micromolar and picomolar range [86,163]. More recently, syntheses of pyrazole (1,2-diazole) derivatives guided by rational design yielded a compound with a K_i_ = 216 nM, which binds with a 5-carbonyl benzimidazole moiety to the S1 pocket extending further to the S4 pocket (Figure 6B) [164]. However, despite many efforts, none of these compounds gained pharmacological significance.

From a therapeutic perspective, the abundant and, in the case of KLK2 and KLK3/PSA, exclusive prostate-localization with defined substrate preferences make prostatic KLKs attractive targets for precision medicine. In recent years, KLK-activated prodrugs and immunotherapies have become the dominating strategies under investigation. The idea to engineer prodrugs exploiting semenogelin recognition sequences was introduced in 1997 and realized one year later with the Mu-HSSKLQ↓L-doxorubicin construct, which released the cytotoxic Leu-dox moiety upon KLK3 cleavage [165,166]. To name a few, similar constructs were generated with PSA cleavage sites of the cytotoxic 5-fluorodeoxy-ridine, desacetyl-vinblastine, the pore forming pro-aerolysin, an N-(2-hydroxypropyl) methacrylamide copolymer, cyclopamine, phosphoramide mustard, or the alkaloid emetine [167,168,169,170,171,172]. Other studies used albumin as a drug carrier, binding EMC-RSSYYSL-PABC-paclitaxel (taxol, EMC ε-maleimidocaproyl, PABC p-aminobenzyloxy-carbonyl) or related constructs with cabazitaxel, which is already an approved drug for chemotherapy of PCa [173,174]. For more than 20 years, the natural apoptosis inducing compound thapsigargin (TG) from the plant *Thapsia garganica* or “deadly carrot” and its derivatives have been promising candidates to treat PCa (Figure 6C) [175]. The TG derivative 12ADT with a 12-aminododecanyl linker can be adapted with oligopeptides as highly specific recognition sequences for KLK2 and KLK3. Due to the localization at PCa tumor cells, proteolytic cleavage by these KLKs results in release of the cell penetrating TG moiety that causes a strong Ca^2+^ influx and eventually cell death. Even other trypsin-like serine proteases can be turned into prodrugs, such as an engineered zymogen of granzyme B (GZMB), which comprises the N-terminal propeptide KGISSQY and the catalytic domain of the cytotoxic GZMB [176]. Nevertheless, none of these approaches had successful clinical trials.

Thus, antibody-based radiotherapies and recent progress in immunotherapies seem to the most promising therapeutic approaches for PCa, in particular PROVENGE (Sipuleucel-T) and PROSTVAC involving PSA as the target of vaccination, which both reached phase three clinical trials [177]. However, the preferred target of imaging and therapies is the prostate-specific membrane antigen (PSMA, M28.010), not to be confused with PSA, a Zn^2+^ dependent carboxypeptidase, which cleaves at P1-Glu↓P1′-Glu residues [178]. In 2022, the FDA approved PLUVICTO, which exploits the high expression of PSMA on PCa tumor cells and the binding capacity of the radioligand ^177^Lu-PSMA-677, which kills cells by β-radiation. Among the immunotherapies, the bispecific IG1 antibody pasritamig was investigated in a phase I clinical trial study with 174 patients. It binds both CD3 receptors on T cells and KLK2 on the surface of tumor cells and exhibits significant anti-tumor activity without adverse effects [179]. Seemingly, pasritamig has entered phase III clinical trials [180]. Other efficient therapeutic approaches employ bispecific T cell engagers (BiTEs, e.g., JNJ-78278343 for KLK2), autologous chimeric antigen receptor T cells (CAR-T), e.g., JNJ-75229414, whereas antibody–drug conjugates, cyclotide-based selective inhibitors for KLK4, and peptide/aptamer-based inhibitors are not neglected [138]. The Sipuleucel-T, CAR-T cell, and BiTE approaches are either based on harvesting patient cells, or blood and tumor samples, each resulting in T-cell mediated elimination of tumor cells [181]. Sipuleucel-T was approved by the FDA as a vaccine targeting the prostatic acid phosphatase (PAP), an antigen found in PCa. Additionally, KLK-derived epitopes showed promising results in inducing CD4^+^ and CD8^+^ T cell responses, supporting their use in cancer immunotherapy [182]. Since KLK2 is significantly expressed on surfaces of localized and metastatic hormone-sensitive PCa cells, corresponding immunotherapeutic tools were developed [183]. Accordingly, KLK2 was targeted with bispecific T-cell engagers that resulted in tumor cytotoxicity and with an α-radioconjugate ^225^Ac-anti-KLK2-mAb, which had anti-tumor activity in a xenograft model, similar to CAR-T cells.

**Figure 7:**
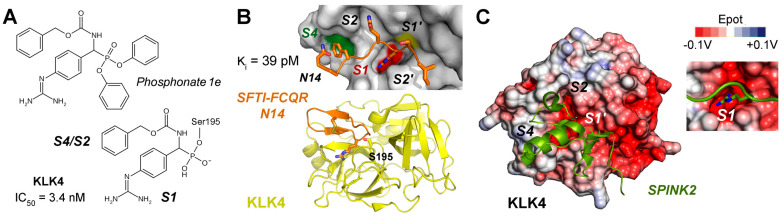
(**A**) A covalent but reversible phosphonate inhibitor of KLK4 with a guanidinophenyl group as P1 analog has an IC_50_ of 3.4 nM. Two phenoxy groups leave upon the Ser195 nucleophilic attack on the electrophilic phosphorus. The carboxybenzyl group (Cbz) may bind to the S2–S4 pockets. (**B**) The engineered variant of the cyclic sunflower trypsin inhibitor SFTI-FCQR-Asn14 replacing the natural sequence RCTK was highly potent with a K_i_ = 3.89 pM. It binds like a substrate to the S2′ to S4 pockets, which are negatively charged (red), hydrophobic (green), or mixed polar and hydrophobic (white), with the catalytic S195 shown as yellow patch. SFTI is stabilized by a disulfide and three Pro and its short β-sheet aligns with an extended β-sheet of KLK4. (**C**) The engineered Kazal-type inhibitor SPINK2 with the VCQR motif exhibited a K_i_ of 160 pM for KLK4 (PDB 6KBR).

KLK4 (S01.251) and KLK11 (S01.257) are both tryptic proteases with a preference for propeptides of related pro-KLKs, such as SR↓IVGG [184]. However, with respect to profiling studies and natural substrates, both KLKs have a mixed specificity accepting P1-Gln, Tyr, or Met residues [185,186]. Since functional and structural data on KLK11 are scarce, it will be further discussed in Section 4 on AI-predicted structures. Apart from its role in prostate, KLK4 is, together with matrix metalloproteinase 20 (MMP-20), crucial for the development of the hardest layer of teeth [187]. Degradation of their substrates ameloblastin, amelogenin, and enamelin is required for the formation of enamel crystallites, which resemble the mineral hydroxyapatite. Using the MEROPS specificity matrix, a consensus sequence for KLK4 substrates would be RQKR↓SLGG and it could be a very efficient activator of the other prostatic KLKs [188]. Unlike most other KLKs, KLK4 was rarely studied in a systematic manner as a target of small molecule inhibitors. Exceptionally, a highly potent phosphonate inhibitor (1e) with a guanidinophenyl group as P1 analog was discovered exhibiting an IC_50_ of 3.2 nM (Figure 7A) [189]. Moreover, inhibitor 1e was more than 70-fold specific with respect to KLKs 1, 2, and 8, as well as to uPA, tPA, plasmin, thrombin, and FIXa. More often, naturally occurring inhibitors and their engineered variants were investigated. For example, SPINK6, a Kazal-type inhibitor, has a K_i_ of 27 nM and binds KLK4 in a substrate-like manner, due to its shape complementarity and positively charged surface, which is interacting with a negatively charged region of KLK4 [190,191]. In particular, engineered variants of the cyclic sunflower trypsin inhibitor like SFTI-FCQR and SFTI-FCQR-Asn14 turned out to be highly potent and selective with K_i_ values of 3.89 nM and 39 pM, respectively [192]. Wild-type SFTI-1 exhibits a P1-Lys5 and is stabilized by the internal disulfide Cys3-Cys11, whereby Cys3 is the P3 residue by coincidence. Crystal structures of both complexes were determined (PDBs 4K1E and 4KEL) at very high resolution (Figure 7B) [193,194]. MoCoTI-II is a 34-residue cyclic peptide with two disulfides from the plant *Momordica cochinchinensis* and its FCQR variant inhibited KLK4 with a K_i_ of 100 pM [195]. Engineered variants of the Kazal-type SPINK2 inhibitor exhibited for the corresponding VCQR and CCQR motifs had K_i_ values between 160 and 210 pM [196]. The latter SPINK2 variant was solved as complex with KLK4 and suggested as therapeutic biomolecule (Figure 7C). Eventually, the crystal structure of KLK4 with the Kunitz-type inhibitor BbKI and several variants from the plant *Bauhinia bauhinioides* were determined, whereby the wild-type inhibitor had the lowest K_i_ of 45 pM [197]. Two monoclonal antibodies raised against KLK4 were already mentioned in Section 2.6 [77]. Remarkably, according to molecular docking, the binding epitope of the mAb ABS4 comprises mostly the 75-loop with its cation binding site, which was shown to be crucial for Zn^2+^ inhibition of the KLK4 protease [198].

In conclusion, KLK2, KLK3, KLK4, KLK5, KLK11, and possibly KLK15 are important for normal prostate physiology but become reprogrammed in prostate cancer to drive tumor progression. Their enzymatic roles, regulatory mechanisms, and accessible extracellular location provide unique opportunities for biomarker development and targeted therapy in both hormone-sensitive and castration-resistant PCa. Apart from the roles of KLK11 in prostate, a presumably pathogenic variant of the *KLK11* gene appears to be one cause of Mendelian disorders of cornification (MeDoC), which is a group of heterogeneous skin conditions of hyperkeratosis and dysregulated scaling [108].

### 3.3. Skin-Derived KLKs 5, 7, and 14

KLKs 5, 7, and 14 are abundantly secreted by keratinocytes in the upper epidermis, particularly in the stratum granulosum (SG) and stratum corneum (SC). Together, they form a proteolytic cascade essential for corneodesmosome degradation, enabling physiological desquamation and maintaining the skin barrier [199]. Thereby, these KLKs regulate inflammation and lipid metabolism in skin by degrading lipid-processing enzymes, they activate protease-activated receptors PAR-1, PAR-2 or PAR-3 on keratinocytes and fibroblasts, and they process antimicrobial cathelicidins and pro-inflammatory cytokines [200,201]. Additional players in these proteolytic systems are KLK6 and KLK8. Collectively, these enzymes target key corneodesmosomal proteins, including the cadherins desmoglein 1 (DSG1) and desmocollin 1 (DSC1), as well as corneodesmosin (CDSN), while KLK14 additionally degrades desmoglein isoforms such as DSG3 and DSG4. Interestingly, these cadherin substrates all exhibit acidic pI values, ranging from 4.4 to 4.8, which suggests an interaction with the positively charged surfaces of skin-derived KLKs. Once corneodesmosomes, which are specialized desmosomes representing the strongest type of cell adhesion complexes, are degraded in a coordinated manner, corneocytes detach from the outer SC [202].

Regulation of the skin KLK cascade is critical to prevent premature barrier loss. Their major regulator is the endogenous multidomain lymphoepithelial Kazal-type-related inhibitor LEKTI-1, encoded by the *SPINK5* gene. These KLKs are finely tuned by multiple inhibitors, mostly LEKTI-1 or SPINK5, secretory leukocyte protease inhibitor (SLPI), elafin, as well as by pH and Zn^2+^ concentration to ensure epidermal homeostasis [97]. Upon processing of LEKTI-1, inhibitory domains bind and inactivate KLK5 and KLK14 with high affinity, and KLK7 to a lesser extent [96]. Additional inhibitors include SPINK6 and SPINK9 (LEKTI-2), which is particularly relevant in palmoplantar skin, elafin, and α_2_-macroglobulin-like 1 [203,204]. Metal ions such as Zn^2+^ and Cu^2+^ provide reversible suppression, and a pH gradient across the epidermis ensures that significant KLK activation occurs only in the upper SC, with a pH between 4.5 and 5.5. Dysregulation of KLK activity is the major cause of several skin disorders. In particular, atopic dermatitis is among the most widespread inflammatory skin diseases, affecting primarily children and overall about 20% of all individuals [205]. Increased expression and protease activity in atopic dermatitis lesions is known for KLK5 and KLK7, as well as for KLKs 6, 8, 10, 11, 13, and 14. Since KLKs 5, 6, and 14 can activate protease-activated receptor 2 (PAR-2), one can assume that the subsequent signaling results in the typical inflammation and itching [206]. In the rather rare genetic disease Netherton Syndrome (NS), which is found in about 1 of 200,000 newborns, *SPINK5* mutations cause the loss of functional LEKTI-1, leading to uncontrolled KLK activation, excessive corneodesmosome degradation, skin barrier collapse, and inflammation via PAR-2 signaling [207]. Besides atopic dermatitis and NS, overactive KLKs are implicated in psoriasis and rosacea, in which abnormal proteolysis disrupts barrier homeostasis and modulates the processing of antimicrobial cathelicidins to release peptides such as LL-37 [208].

**Figure 8:**
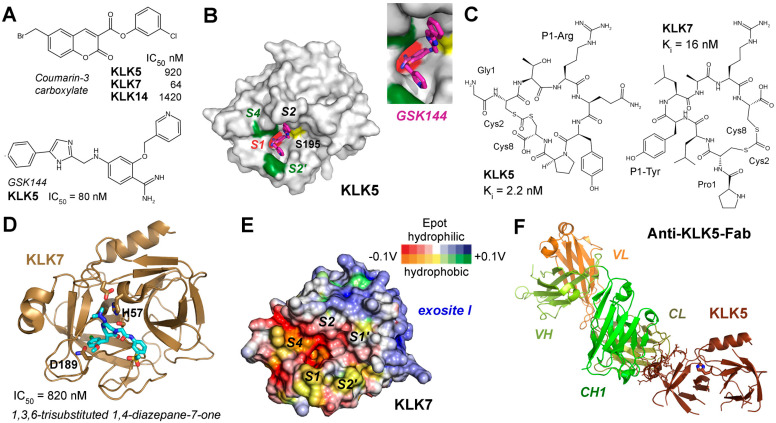
Inhibitors and structures of skin-derived KLKs. (**A**) A series of coumarin-3-carboxylate derivatives yielded specific inhibitors for KLKs 5, 7, and 14 (upper panel). Below the phenylimidazole GSK144 is shown, which inhibited KLK5 with a low nM IC_50_. (**B**) The crystal structure of the complex KLK5-GSK144 complex revealed the binding mode: the phenylimidazole binds the S2 pocket and a pyridinylmethoxy benzimidamide group binds the S1 pocket (PDB 6QFE). Hydrophobic zones on the molecular surface are depicted as green patches and the catalytic Ser195 is shown as yellow patch. (**C**) Two cyclic octapeptides from a phage display study exhibited highly specific inhibitors of KLK5 with a P1-Arg and for KLK7 with a P1-Tyr. (**D**) Using a lead from a previous study, a series of 1,3,6-trisubstituted 1,4-diazepane-7-ones resulted in KLK7 inhibitors with IC_50_ values below 1 µM. The X-ray structure confirmed the binding of a chloro-methoxyphenyl group in the S1 pocket (5YJK). (**E**) Similar to KLK5 and KLK14, KLK7 exhibits a positively charged region, which corresponds to the one of thrombin. Polar and hydrophobic regions are depicted according to their electrostatic potential (Epot) from −0.1 V to +0.1 V in red, white (neutral), and blue or orange, yellow (neutral), and green, respectively. (**F**) A bispecific anti-KLK5/7 Fab antibody reduced inflammation in skin disease models and bound to KLK5 from residues 164–178. Surprisingly, the whole active site region transformed to a super-zymogen state, with undefined disordered stretches of residues 16–25, 37–42, 58–62, 68–78, 90–98, 140–156, 186–196, and 215–221.

Most likely, KLK5 (S01.017) prefers to cleave after P1-Arg/Lys and acts as the primary initiator of the cascade, activating itself and, subsequently, cleaving and activating pro-KLK7 and pro-KLK14 [209]. KLK7 (S01.300) displays chymotrypsin-like activity that accepts aromatic P1 residues, i.e., Tyr and Phe, while KLK14 (S01.029) is predominantly trypsin-like but can occasionally cleave in a chymotrypsin-like manner after P1-Tyr, reinforcing the activation loop and broadening substrate specificity [210]. From 2011 onwards, several studies investigated plant isocoumarins, synthetic isomannide, 3-acyltetramic acids, 1,2,4-triazoles, coumarins, pyrido-imidazodiazepinones, trisubstituted 1,4-diazepan-7-ones, and imidazolinylindoles as inhibitors of the skin KLKs [97]. An in silico screening of the ChemBridge database with more than 600,000 compounds yielded about a dozen skin KLK-targeting inhibitors [211]. They all have in common that they inhibit at least one of these KLKs in the low micromolar or even nanomolar range and exhibit some selectivity but are not further developed as lead compounds. A study of about 60 coumarin-3-carboxylate derivatives produced nanomolar inhibitors for KLKs 5, 7, and 14, whereby in case of KLK7 a suicide mechanism by attacking Ser195 was very likely, although according to docking studies, none of the compounds bound to the specificity pockets in the active site cleft (Figure 8A) [212]. Structure-functional investigations based on rational design on crystal structures of KLK5 revealed the binding mode of the GSK144 inhibitor with an IC_50_ of 80 nM, whereby its phenylimidazole moiety occupied the S2 pocket and a pyridinylmethoxy benzimidamide group occupied the S1 pocket (Figure 8A,B) [213]. Similarly, the covalent but reversible boronate compound GSK951 inhibited KLK5 in vitro with an IC_50_ of 250 pM and significantly in skin samples of NS patients [214]. Moreover, it possesses a 100-fold selectivity for KLK5 over KLK7 and KLK14. The supplementary material of this publication refers to the paper by Thorpe and coworkers on GSK144 exhibiting the same S1 binding pyridinyl-methoxy benzimidamide group in a cocrystal structure complex of KLK6 mutants as KLK5 surrogates [213]. Several coumarinic esters inhibited KLK7 in the nanomolar range, whereby compound 2 alkylated the catalytic His57 with an IC_50_ of 65 nM was observed in two alternate conformations in the crystal structure (PDB 6SHI), occupying either the prime side or the non-prime side pockets [215]. Two patents of the Novartis AG describe potent KLK7 inhibitors: one is a peptidic proline derivative linked to aromatic heterocycles with an IC_50_ of 3 nM (US2010/0256144A1), the other one is a cyclic depsipeptide comprising several canonical and non-canonical amino acids exhibiting an IC_50_ of 200 pM (US2009/0156472A1). Depsipeptides occur in nature and possess at least one ester instead of an amide group. Similarly, the screening of several billion cyclic peptides in a phage display study yielded after elimination of potential protease recognition sites two highly potent inhibitors for KLK5 with a K_i_ of 2.2 nM and for KLK7 with a K_i_ of 16 nM (Figure 8C) [216]. There are more studies on inhibition of skin-derived KLKs; however, we did not discuss those investigating murine proteins or mixtures of human and murine proteins. Nevertheless, a series of 1,3,6-trisubstituted 1,4-diazepane-7-ones was investigated as novel human and murine KLK7 inhibitors, of which several ones inhibited with IC_50_ values in the nanomolar range and were deposited in the PDB (Figure 8D) [217,218].

Two studies engineered the SFTI-1 derivatives directed against KLKs 5, 7, and 14, resulting in several quite specific variants, which inhibited even in the low nanomolar range [219,220]. Further optimization of the SFTI-1 variants resulted in a distinct set of inhibitors with P1-Arg residues that targeted KLK5 and KLK14 with at least 10-fold specificity and P1-Phe variant with extraordinary specificity for KLK7 with a K_i_ of 140 pM [221]. The synthetic LEKTI-1 domain d6 inhibited KLK5 with an IC_50_ around 125 nM like its recombinant counterpart in a reversible manner [222]. Also, serpinA12 (MEROPS I04.091) or vaspin seems to be a very specific, irreversible inhibitor of KLK7 and KLK14, whereas it does not inhibit KLK5 [223]. Thus, it may be a starting point for therapeutic approaches. Based on a phage display study on the specificity of KLK14, a series of serpin inhibitors was generated, with a P1-Tyr in the reactive center loop of α1-antichymotrypsin being the most specific one [224]. Other biomolecules may bind the positively charged region near the prime side specificity pockets, which was observed in KLK5 and KLK7 (Figure 8E). A monoclonal bispecific human anti-KLK5/KLK7-Fab antibody improved skin barrier function and reduced inflammation in mouse models of NS and atopic dermatitis [75]. Intriguingly, this Fab bound KLK5 rom residues 164 to 178 distant from the active site region, which was completely disordered, suggesting an allosteric inhibition mechanism (Figure 8F). Inhibition of KLK activity not only stabilizes the barrier but also limits pro-inflammatory signaling, making these proteases attractive drug targets for barrier-associated dermatoses [225]. Since KLKs play distinct roles in skin cancer, e.g., KLK6 and KLK7 are upregulated in cutaneous melanoma, they may represent future targets for corresponding therapies. By contrast, KLK14 was found at elevated protein levels detected by cytoplasmic staining in basically all types of cancer and, thus, may become a “magic” target in cancer therapies (https://www.proteinatlas.org/ENSG00000129437-KLK14/cancer, accessed on 10 December 2025).

In summary, KLK5, KLK7, and KLK14 operate as an integrated proteolytic module in the epidermis, with tightly controlled activation and inhibition ensuring effective desquamation without compromising barrier integrity. Therapeutic approaches targeting KLKs are emerging, including monoclonal antibodies against KLK5 and KLK7, or engineered peptide inhibitors, such as SFTI, or Kazal-type and serpin inhibitors of KLKs 5, 7, and 14, or their modulation through metal ion supplementation. Their central role in skin physiology, combined with their contribution to inflammatory skin diseases when dysregulated, underscores the therapeutic potential of precisely modulating KLK activity.

### 3.4. Brain- and Neuron-Associated KLKs 6, 8, 9, and 10: Roles in Physiology, Pathology, and Natural Inhibition

Kallikrein-related peptidases (KLKs) 6, 8, 9, and 10 are expressed in various cell types of the central nervous system (CNS), including neurons, astrocytes, and oligodendrocytes [226]. Apart from its crucial role in the outer layers of skin, KLK7 participates with these KLKs beyond their established functions in peripheral tissues. They regulate extracellular matrix (ECM) remodeling, synaptic plasticity, peptide processing, and neuroinflammatory signaling. Expression is dynamic and influenced by neuronal activity, age, and disease state, implicating them in both CNS homeostasis and pathology [227].

KLK6 or neurosin is enriched in oligodendrocyte-rich white matter and contributes to myelin maintenance, axon–glia communication, and oligodendrocyte survival via protease-activated receptor (PAR1/PAR2) signaling [228]. Also, KLK7 is expressed in neurons and glia, participates in ECM turnover, and can directly cleave the plaque-forming amyloid-β (Aβ) peptides of 36 to 43 residues length, which potentially supports neuroprotection and prevents Alzheimer’s disease (AD) [229]. Recent data from studies of wild-type and knockout mice suggest a role of Klk8 in the myelin metabolism of oligodendrocytes [230]. Although KLK8 or neuropsin is expressed only at significant protein levels in specialized areas of the brain, it is very important for synaptic plasticity and memory formation, while genetic variations in SNPs are linked to bipolar disorder, but not to schizophrenia [102,104]. Intriguingly, KLK8 levels vary at several stages of diverse brain regions in fetal and adult development, being most intense in the cerebellum, hippocampus, and cortex [231]. Overexpression in mouse models resulted in spatial memory impairment, whereas KLK8 dysregulation in general is linked to depression in humans. As KLK8 is expressed in hippocampal and cortical neurons, it modulates synaptic connectivity and learning by cleaving cell adhesion molecules such as L1CAM and NCAM [232,233]. KLK9, less studied in the CNS, is detected in specific neuronal populations and may modulate proteolytic cascades relevant to synaptic maintenance [106]. Beyond the CNS, KLK9 can be induced in neutrophils upon cytokine stimulation, pointing to an immunomodulatory function within inflammatory settings [234]. Importantly, KLK9 participates in protease networks within the KLK family and has been implicated in the activation of KLK10, thereby linking its activity to broader biological processes that extend into immunity and cancer biology [235]. KLK10 is expressed in both neuronal and glial compartments and is thought to influence neuroinflammatory regulation and tumor microenvironment remodeling [236]. Expression of KLK10 in tonsils and neutrophils further supports its role in immune regulation [105]. Functional studies reveal that KLK10 can activate CD4^+^ T cells and drive M2 macrophage polarization in colorectal cancer, thereby shaping the immune landscape in a tumor-promoting direction [237]. Furthermore, KLK10-derived peptides have been identified in both HLA class I and class II complexes in ovarian cancer, underscoring their immunogenic potential and relevance as a source of tumor-associated antigens [238].

In cancer biology, KLK10 has been associated with both tumor-suppressive and tumor-promoting roles, depending on the cellular context. On one hand, it can suppress tumor growth via inhibition of the PI3K/Akt/mTOR pathway [239]. On the other hand, in the presence of oncogenic KRAS, KLK10 promotes tumor progression through the PAR1/PDK1/Akt axis, and it has also been linked to epithelial–mesenchymal transition via activation of the FAK/SRC/ERK pathway [240,241]. This duality emphasizes its complex role as a context-dependent regulator of tumorigenesis. Beyond influencing tumor growth, KLK10 has emerged as a critical mediator of drug resistance. In gastric cancer, it promotes resistance against the pharmaceutical monoclonal antibody trastuzumab resistance through the PI3K/Akt pathway, while in breast cancer, it not only contributes to trastuzumab resistance, but also predicts tamoxifen resistance [242,243,244]. These findings establish KLK10 as both a biomarker for therapeutic response and a potential target for strategies aimed at overcoming resistance.

In Alzheimer’s disease (AD), KLK6-8 and 10 have been suggested as AD biomarker candidates, with CSF studies indicating elevated KLK6 and KLK10 in biomarker-confirmed cases, while KLK8 remains largely unchanged and KLK7 is decreased [107,236,245]. KLK6 participates in cleavage of the non-amyloidogenic amyloid precursor protein (APP) with up to 770 residues length, but can also disrupt Aβ42 aggregation, while KLK7-mediated Aβ cleavage supports amyloid clearance [229,246]. KLK8 dysregulation impairs synaptic plasticity, contributing to a cognitive decline [247]. KLK9 and KLK10 have been associated with altered expression patterns in neurodegeneration and gliomas, potentially impacting neuronal survival and glial signaling [226]. In multiple sclerosis (MS) and its experimental autoimmune encephalomyelitis model (EAE), KLK6 secretion by oligodendrocytes promotes demyelination and neuroinflammation [248]. Neutralizing KLK6 activity results in attenuation of inflammatory demyelination [249]. KLK8 is upregulated under inflammatory conditions, suggesting a contributory role in synaptic and structural damage [250]. Data on KLK6, KLK7, KLK8, and KLK10 in MS are limited, but their regulation in neuroinflammatory contexts indicates possible synergistic or modulatory effects within the CNS protease network [251].

Endogenous regulation of KLK activity is mediated by serine protease inhibitors such as α1-protease inhibitor (α1-antitrypsin, AAT, APPI), α1-antichymotrypsin, secretory leukocyte protease inhibitor (SLPI), and kallistatin [11]. In particular, AAT appears to be the major inhibitor of KLK6 in body fluids, such as cerebrospinal fluid, serum, and ascites of ovarian cancer patients [252]. These inhibitors control proteolysis under physiological conditions, preventing excessive ECM degradation, synaptic disruption, or demyelination. AAT is particularly noteworthy for dual activity against KLK6 proteolysis and Aβ42 aggregation, representing a potential therapeutic lead for AD [246]. Loss or imbalance of inhibitor activity in disease states can facilitate unrestrained KLK activity and exacerbate pathology.

CNS-derived KLKs comprise three with primarily trypsin-like specificity and two with chymotrypsin-like specificity. The enzymatic properties of KLK7 were already covered in the section on skin KLKs and KLK9 will be discussed in Section 4. KLK6 (S01.236) exhibits some specificity in the prime side subsites and according to a positional scanning study of combinatorial libraries (PSSCL) for the non-prime side, a consensus cleavage site could be RR↓SAGG, which might be biased by KLK propeptide substrates [185]. Furthermore, KLK8 (S01.244) was investigated with a combined PSSCL and proteomic-based study comprising specificity sites P6 to P6′, resulting in recognition sequences like TKLR↓SILL [13]. The enzymatic activity of KLK10 (S01.246) was hardly investigated, since it is only active with an unusual N-terminus starting with Leu13 and adopts a zymogen-like structure, while its specificity is slightly mixed as it can cleave after P2-Asp-P1-Arg/Met [185,253].

**Figure 9:**
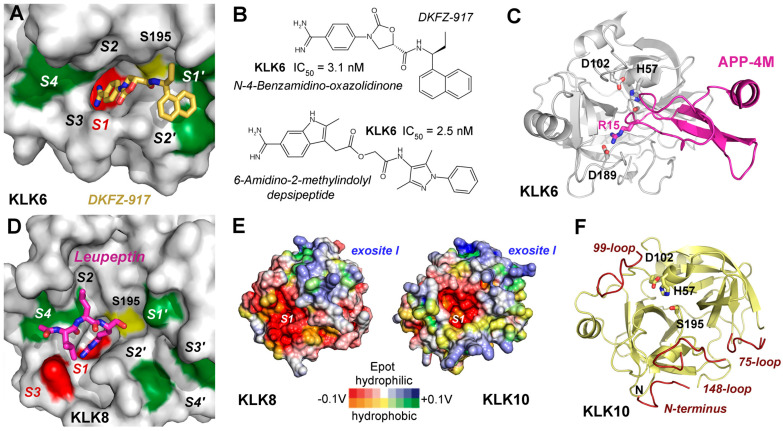
Inhibitors and structures of brain-derived KLKs. Additional structural representations of KLK6 and KLK8 are shown in Figure 1B,C. (**A**) A high-throughput screening (HTS) study resulted in optimized N-4-benzamidino-oxazolidinones with one amide bond, of which DKFZ-917 was the most potent inhibitor of KLK6 with an IC_50_ of 3.1 nM. According to a crystal structure (7QHZ) the inhibitor binds the S1 specificity pocket (red) and extends with ethyl and naphtyl groups to the S1′ and S2′ pockets. The green patches are hydrophobic, the red one is negatively charged, and the yellow patch indicates the catalytic Ser195. (**B**) Structural formulas of DKFZ-917 and of a 6-amidino-2-methyl-indolyl depsipeptide with an IC_50_ of 2.5 nM. Depsipeptides are peptides with at least one ester bond instead of an amide, while docking studies suggest that the latter compound binds from the S1 to the S3′ pockets. (**C**) Complex of KLK6 and the engineered inhibitory Kunitz-type domain APP-4M with a K_i_ of 140 pM, exhibiting four mutations and a P1-Arg15. The catalytic triad residues and the specificity-defining Asp189 at the bottom of the S1 pocket are depicted as sticks, whereas residues 217 and 218 were omitted in the 220-loop for clarity. (**D**) KLK8 in complex with leupeptin, which binds to the non-prime side from the S4 to S1 pockets (5MS3). The X-ray structure confirmed the binding of an arginiyl group in the S1 pocket. Color scheme as in Figure 9A. (**E**) Both KLK8 and KLK10 (AlphaFold model) surfaces possess positively charged regions, which correspond to exosite I of thrombin. Polar and hydrophobic regions are depicted according to their electrostatic potential (Epot) from −0.1 V to +0.1 V in red, white (neutral), and blue or orange, yellow (neutral), and green, respectively. (**F**) KLK10 resembles a zymogen with disordered stretches of residues 13–20 (N-terminus), 71–77 (75-loop), 95–96 (99-loop), and 146–152 (148-loop) shown in red. This state may depend on the elongated N-terminus starting with residue Leu13, which is required for enzymatic activity. However, the catalytic triad is properly formed in an active conformation and can be inhibited by direct binding of Zn^2+^.

Classical small molecule inhibitors with amidinothiophene and *para*-amidobenzylamine as P1 analogs were characterized by Liang and coworkers [254,255]. Compounds **3** and **9** in the respective studies inhibited KLK6 with IC_50_ values of 2.9 µM and 1.8 µM and were structurally characterized (PDBs 3VFE, 4D8N). Another rational design study of *para*-amidobenzylamines found many inhibitors of KLK6 and related proteases exhibiting nanomolar inhibition constants [256]. Using HTS of 350,000 compounds, N-4-benzamidino-oxazolidinones were discovered, with the most potent one inhibiting KLK6 at a pIC_50_ of 8.6, which corresponds to an IC_50_ of 2.5 nM [257]. After an optimized synthesis, the now DFKZ-917 called compound was reported to have an IC_50_ of 3.1 nM and the complex structure with KLK6 was solved (PDB 7QHZ, Figure 9A,B) [258]. Unusual depsipeptides with a 6-amidino-2-methyl-indolyl group as P1-analog acylated the catalytic Ser195 of KLK6 and remained stable for many hours, whereby an IC_50_ of 2.5 nM was observed for compound 39, a derivative of the DFKZ-251 scaffold (Figure 9B) [259]. Deferasirox derivatives, being 1,2,4-triazoles with two hydroxyphenyl groups, were characterized as Fe^3+^-enhanced non-competitive and uncompetitive inhibitors of KLK6 and KLK8 with K_i_ values around 10 µM [136]. Since SPINK6 is expressed in the brain and inhibits KLK6 with a K_i_ of 140 nM, it was suggested as a physiological regulator [190]. The amyloid precursor protein contains a functional Kunitz inhibitor domain from residues 29–341, which was engineered by four mutations to APP-4M, being a highly potent KLK6 inhibitor with a K_i_ of 140 pM (Figure 9C) [260]. Preclinical trials of APP-4M and the related variant APP-3M were promising with cell culture and mouse models for ovarian cancer and breast or prostate cancer [70]. KLK6 inhibition represents a promising approach for promoting remyelination and reducing neuroinflammation in MS [261].

Enhancing KLK7 activity could improve Aβ clearance in AD, while selective modulation of KLK8 may help restore synaptic integrity [262]. Also, miRNA-based inhibition of overexpressed KLK8 in an AD mouse model enhanced neuroplasticity, which may pave the way for therapies in humans [263]. Otherwise, no relevant studies on inhibitory compounds or biomolecules for KLK8 with therapeutic potential were found in the literature. Therefore, the published structures of KLK8 as complex with the aldehyde inhibitor leupeptin and ligand-free remain just a starting point for pharmaceutical research, which may take into account the stimulatory Ca^2+^ and the inhibitory Zn^2+^ binding sites (Figure 1C and Figure 9D) [13]. KLK8 and KLK10 (AlphaFold model) exhibit negatively charged regions around their S1 pockets, while both have a positively charged surface region similar to the exosite I of thrombin (Figure 9E). KLK9 and KLK10, though less characterized, may contribute to multi-target CNS protease strategies. Perhaps, it is required to find new types of inhibitory compounds that target KLK10 with its zymogen-like structure, which may depend on regulators or a special substrate as inducer of the active protease conformation (Figure 9F) [253]. Therapeutic designs mimicking natural inhibitors or employing selective protease modulators could fine-tune activity while preserving essential CNS functions. KLK-based biomarker panels, integrating KLK6–KLK10 measurements in CSF or plasma, may support early diagnosis and prognostic assessment in neurodegenerative and demyelinating disease [107,264].

### 3.5. KLK12, KLK13, and KLK15: Emerging Insights from Cancer Dysregulation

Among the kallikrein-related peptidases, KLK12, KLK13, and KLK15 with trypsin-like specificity represent members whose physiological functions remain less well defined than those of other family members. Nevertheless, dysregulation of their expression and activity has been consistently reported in cancer and other diseases, suggesting that their role in the malignant transformation could shed light on their biological function. As with other KLKs, these proteases have been associated with tumor growth, invasion, immune regulation, therapy resistance, and prognosis, underlining their potential as both biomarkers and therapeutic targets [86]. Expression of KLKs 12, 13, and 15 is variable across tissues and tumor types, and their regulation occurs at multiple levels, including epigenetic silencing and hormonal control, whereby KLK15 stands out [21,265]. In particular, human neutrophils show high levels of KLK13 and KLK15 mRNA, while KLK12 can be detected by immunostaining as well, but only KLK13 is strongly induced by cytokines [234]. Although the extended substrate specificity from the S4 to S4′ pockets of all three KLKs 12, 13, and 15 is well characterized according to the MEROPS database (https://www.ebi.ac.uk/merops/index.shtml, accessed on 10 December 2025), no peptidic inhibitors have been designed so far and data on library screening of natural or synthetic compounds are lacking in the relevant literature. In the case of KLK12 (MEROPS entry S01.020) and KLK13 (S01.306), just 20 reported cleavages may be a small number as about 30 should suffice to determine the corresponding specificity from cleavage entropies [266]. By contrast, the hundreds of cleavages determined by MS for KLK15 (S01.081) level out all specificity information from S4 to S4′ except for the overwhelming P1-Arg [114].

KLK12 is expressed in endothelial cells and has been linked to angiogenesis-associated proteolysis [109,267]. Thus, KLK12 has been implicated in the regulation of endothelial cell behavior through AMPK/mTOR signaling, influencing processes such as fibronectin polymerization and angiogenesis [268]. KLK12 protease is a remarkable exception among the KLKs, since to date no protein expression has been reported for most cancer tissues, except for breast cancer (Table 3) [116]. However, it has been proposed as a potential therapeutic target in colorectal cancer on the mRNA level and its expression was silenced by siRNA linked to selenium nanoparticles, which may be a groundbreaking approach [269]. Since KLK12 efficiently cleaves the haemagglutinin (HA) precursor H1 and H2 subtypes of influenza viruses, allowing for membrane fusion and endocytosis with host cells, another pharmacological field could be opened [110]. This pathophysiological role is very likely, as KLK12 and KLK5, which preferentially cleaves the H3 subtype of HA, are not only present in the respiratory tract but also in nasal wash samples. The KLK12 activity against chromogenic substrates containing a P1-Arg is efficiently inhibited by monoclonal antibodies and the irreversibly inhibiting serpin α_2_-antiplasmin, which may offer routes to engineered pharmaceutical inhibitors, as other engineered serpins are reaching clinical development [270,271]. Also, the high potency of the reversible KLK12 Kazal-type inhibitor SPINK6 with a K_i_ of 1.1 nM may have therapeutic potential, as a mouse model of atopic dermatitis corroborated [190,272]. Kazal inhibitor scaffolds have been proposed for engineering of therapeutic biomolecules [273]. Also, the plasmin targeting tissue factor pathway inhibitor 2 (TFPI-2) inhibits KLK12 with a very low K_i_ of 3.0 nM [274]. KLK13 is expressed in a variety of tissues (Table 1), while epithelial tissues seem to dominate, but also in immune cells such as neutrophils, suggesting both structural and immunoregulatory functions [275]. Regarding mRNA levels, KLK13 seems to play an important role in head and neck cancer, whereas on the protein level it is only significantly present in prostate, urothelial, and cervical and lung cancer (Table 3). KLK13 is associated with the degradation of ECM components and the promotion of epithelial–mesenchymal transition, supporting invasive behavior [276]. A study with recombinant human KLK13 and various body fluids revealed that it binds to α_2_-macroglobulin, as well as to the serpins α1-antichymotrypsin and α2-antiplasmin, although in vitro inhibition was not near 100% [277]. As KLK13 may play a minor role in desquamation, it was demonstrated that a recombinant LEKTI-1 (SPINK5) construct comprising domains 1–6 inhibited yeast-expressed KLK13 with an IC_50_ of 29 nM [278]. The highly potent SPINK6 inhibited KLK13 with a K_i_ of 280 pM may have therapeutic potential by engineering corresponding biomolecules [190]. Since gingipain cysteine proteases secreted by the bacterium *Porphyromonas gingivalis* degrade SPINK6 as the main inhibitor of salivary kallikreins such as KLK13, cures for peridontitis may be improved [112].

KLK15 possesses the unique Glu189 at the bottom of the S1 pocket, which can strongly shift the selectivity in favor for Lys versus Arg [188]. In vitro assays demonstrated that KLK15 activated its own proform, pro-KLK8, and pro-KLK14 at the highest rates, as they all contain a P1-Lys in the propeptide. As already mentioned in Section 3.2, the KLK15 gene is strongly upregulated in prostate cancer cell lines on the mRNA level, which was essentially confirmed for patient samples [154,279]. Two immunoassay-based studies confirmed that KLK15 is only present in small amounts of adult tissue extracts of colon, prostate, and thyroid, while it was also found in skin, liver, heart, kidney, and in other tissues, in particular, in many fetal ones [5,280]. Another study localized it mainly in testis and suggested a role in spermatogenesis [113]. By contrast, a degradomic analysis for KLK15 showed that ECM molecules such as collagens, laminins, fibronectins, and integrins are preferred targets [114]. In line with these findings, a KLK15 missense variant seems to be the genetic cause of Hypermobile Ehlers–Danlos syndrome (hEDS), a condition characterized by joint hypermobility and chronic pain [115]. A corresponding KLK15 knock-in mouse model showed hEDS features in tendons and heart with altered cytokine levels, indicating a physiological role for KLK15 in ECM-immune system regulation. In contrast to all other KLKs, no inhibitory compound or pharmaceutical approaches concerning KLK15 have been reported so far.

## 4. Future Aspects: AI-Predicted Structures of KLKs Explain Functional Parameters

**Figure 10:**
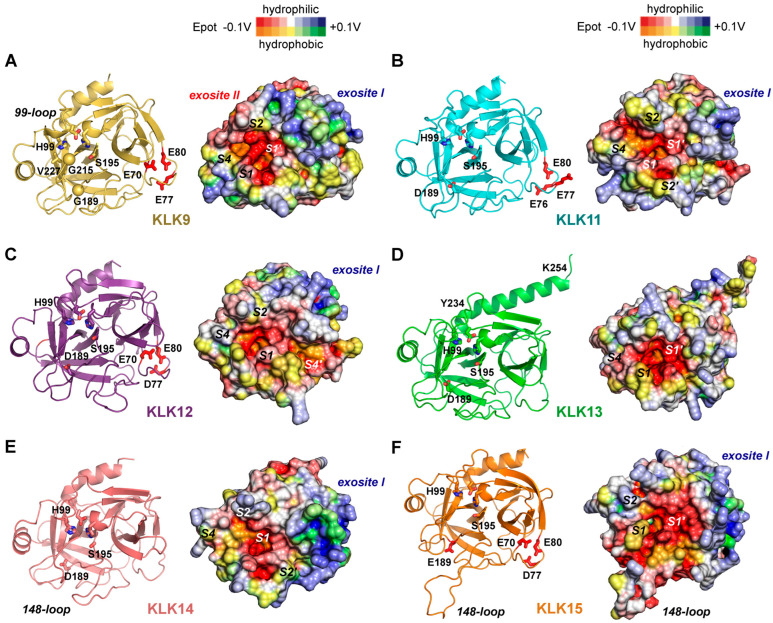
Structural models of KLKs 9 and 11–15 computed by the AlphaFold server. The ribbon representations with sticks and spheres in the left panels have labels for the catalytic S195, the P1-specificity-determining residue 189, and the conserved H99, which separates the S2 and S4 pockets. The molecular surfaces in the right panels were obtained from the eF-surf server of the PDBj using the predicted coordinates. They combine polar regions of negative (red) and positive (blue) electrostatic potential (Epot) from −0.1 V to +0.1 V with hydrophobic patches (orange to green, −0.1 V to +0.1 V), while white and yellow zones are neutral. (**A**) The chymotryptic KLK9 exhibits the unique Gly189 (golden sphere) at the bottom of the S1 pocket. Its 99-loop with a four-residue extension forms an unusual helical turn at the S2 pocket. Replacing the common W215, the unique G215 (golden sphere) cannot form the bottom of the deep mixed polar-hydrophobic S4 pocket where V227 is located. The S1 pocket is overall polar and hydrophobic, whereas the S2 and S1′ pockets are negatively charged. A positively charged region near the prime side region coincides with exosite I of thrombin, while a corresponding potential exosite II is negatively charged in contrast to the one of thrombin. Three Glu residues may constitute a Ca^2+^ binding site similar to trypsin (70, 77, 80) (**B**) The tryptic KLK12 exhibits a negatively charged region from the S3 to the S2′ pockets, whereas the S4 pocket is more hydrophobic. In addition, it may have a small positively charged exosite I and three potential Ca^2+^ ligands (E76, E77, E80). (**C**) Apart from the negatively charged S1, S1′, and S4′ subsites, all other subsites from S4 to S3′ are overall polar or slightly hydrophobic. E70, D77, and E80 are potential Ca^2+^ binding ligands near the positively charged region of an exosite I. (**D**) KLK13 appears to possess a uniquely extended C-terminal α-helix of 21 residues from Y234 to K254 with unknown function. Otherwise, the region from the S1 to S2′ pockets seems rather conventional with an overall negative charge, while the other pockets from S4 to S4′ are mixed polar and hydrophobic. (**E**) Similar to the other skin-derived KLKs 5 and 7, the tryptic KLK14 seems to have an extended positively charged exosite I, while its S1 and S2′ pockets are negatively charged. All the pockets from S4 to S4′ are more hydrophobic or polar, respectively. (**F**) Although the uncommon E189 of KLK15 might confer a preference for P-Lys over P1-Arg residues, functional data could not confirm this assumption yet. The E70, D77, and E80 side-chains may constitute a Ca^2+^ binding site within the extended positively charged exosite I. By contrast, a negatively charged region stretches from the S1′, S1, and S3 subsites to the base of the presumably unstructured 148-loop, which is elongated by a 10-residue insertion. Resembling KLK12, residues E70, D77, and E80 may constitute a Ca^2+^ binding site.

As structural predictions by artificial intelligence become increasingly important, we present coordinates calculated by the online server using AlphaFold3 for KLKs 9, and 11–15 (Figure 10) [281]. All results for the best prediction or” model 0” exhibited overall very high scores over 90 (range from 0 to 100) according to predicted local distance difference test (pLDDT), which is a measure of local confidence per residue [282]. While the original lDDT method includes all atoms of a structural model without any superposition using the correctness of local distance differences, the pLDDT corresponds to a similar Cα test (lDDT-Cα), which yields reliable scores for an estimate of the agreement between prediction and a potential experimental structure within a short computing time. All predicted models were inspected in 3D with the molecular graphics software COOT v0.8.9.2 and represented by PyMOL v0.99 [283,284]. Electrostatic potentials at the molecular surface of modeled KLKs were calculated with the eF-surf server (https://pdbj.org/eF-surf/top.do, accessed on 10 December 2025) [28,285]. Basically all KLKs contain confirmed or putative N-glycosylation sites in the protease domain or even in their propeptides, while the more elusive O-glycosylation does occur as well [286]. The standard output of AlphaFold does not indicate such sites; however, currently progress is being made with the prediction of protein–glycan interactions [287]. Many KLKs are inhibited by the physiologically relevant metal ions Zn^2+^ and Cu^2+^ in the low micromolar range. Among them are KLKs 2, 3, 4, 5, 7, 8, 10, and 14 [24]. Zn^2+^ or Cu^2+^ binding sites were identified in the crystal structures of KLKs 4, 5, 7, and 10 as well as by mutational analyses of KLK2 and KLK8 or modeling studies as in case of KLK3. However, due to the diversity of the binding sites and inhibition types, the KLK models obtained by AlphaFold do not allow for prediction of such binding sites. For KLK12, an IC_50_ near 10 µM Zn^2+^ was reported, while preliminary investigations found IC_50_ values around 1 µM for KLK9 and 100 µM for KLK15, respectively [114,235,270].

Unsurprisingly, AlphaFold placed the unique Gly189 of KLK9 at the bottom of the S1 pocket which is not known for any other chymotrypsin-like serine protease (Figure 10A). The large S1 pocket of this model comprises mixed polar and hydrophobic residues, which could accommodate Phe, Tyr, and Trp residues. Human chymotrypsin B (MEROPS S01.152) and bovine α-chymotrypsin (S01.001) exhibit Ser189 and favor P1-Phe/Tyr over P1-Trp [288]. For KLK9 (S01.307), MEROPS lists a single natural substrate, gurmarin, with the recognition sequence Cys-Ile-Pro-Tyr↓Tyr-Leu-Asp-Cys, while P1-Tyr was about 7-fold preferred over P1-Phe in fluorogenic substrates [235]. In line with this primary P1 specificity, KLK9 was found in complex with α1-antichymotrypsin in ovarian cancer ascites [289]. Similarly, the potentially hydrophobic S2 pocket is shaped by His99 and Leu95C, which is part of a short unusual helical turn of the 99-loop within a four-residue extension. Another feature of KLK9 is the unique Gly215, whereas all other KLKs possess a Trp or Phe in this location. Thus, the bottom of the large and deep S4 pocket is formed by Val227, ready to accommodate aromatic and aliphatic residues. Interestingly, the 70–80 loop exhibits three Glu residues (70, 77, and 80), suitable for divalent cation binding like in bovine trypsin or KLK8 [13,290]. Stimulation of activity by Ca^2+^ binding as in these proteases would also explain the significant increase in KLK9 activity at low mM Mg^2+^ and medium Ca^2+^ concentration [235]. Moreover, KLK9 might have a positively charged exosite I near prime side region like thrombin or KLK5 and KLK7, whereas a putative negatively charged exosite II would confer a novel functionality [186].

In contrast to the strongly negatively charged S1 pockets of several other tryptic KLKs, the AlphaFold model of KLK11 exhibits a deep polar and partially negatively charged S1 pocket, which is still suitable for accommodation of Arg and Lys residues, agreeing with the known specificity (S01.257) [291]. The S2 pocket, the exposed S3 subsite, and the S4 pocket are more polar as well, while the S1′ and S2′ subsites are negatively charged (Figure 10B). By contrast, a small positively charged exosite I is found nearby in the prime side region. The 99-loop is extended by four residues without special features, whereas the 70–80 loop contains three Glu residues (76, 77, and 80) with the potential for divalent cation binding.

As could already be derived from a sequence comparison, KLK12 with tryptic specificity has little unusual features, with the exception of negatively charged spots in the prime side region from about S2′ to S5′ (Figure 10C). A relatively small positively charged exosite I is nearby, while KLK12 possesses the potential divalent cation ligands Glu70, Asp77, and Glu80 in the 70–80 loop. The S2 to S4 pockets are mixed polar and hydrophobic, separated by the conserved His99. According to all reported cleavages, KLK12 (S01.020) slightly prefers P1-Lys over Arg, in line with the significant autoactivation based on a Lys15 in the propeptide [184,270].

The most unusual characteristic of KLK13 is the C-terminal 12-residue extension, which according to AlphaFold forms a long α-helix from Tyr234 to Lys254 (Figure 10D). Besides a four residue longer 99-loop, KLK13 (S01.306) seems to be sort of an average trypsin-like protease without potential exosites or metal binding sites. A specificity profiling study found that P4-Val/Tyr, P3-Arg, P2Leu/Phe/Met, and P1-Arg are the most favored residues [292]. These findings agree very well with the properties of the molecular surface based on the AlphaFold top solution. The S3 to S2′ pockets appear to be negatively charged, while the S4 subsite appears to be mixed polar and hydrophobic.

The sequence of KLK14 (S01.029) reveals no exceptional characteristics, regarding insertions, metal binding sites etc. Besides a clear preference for P1-Arg over P1-Lys, its specificity was the following: P4-Tyr/Trp, P3-Arg/Lys/Ser/Ala/Met, and P2-His/Asn/Ser/Pro/Ala [292]. The molecular surface properties calculated by the eF-surf server concord very well with this specificity profile, as the S4 pocket is more hydrophobic, while the S3 and S2 pockets are more polar (Figure 10E). A negatively charged spot at the S2′ subsite might accommodate basic residues like Arg and Lys. In addition, KLK14 appears to possess an extended positively charged exosite I near the prime side region, resembling those of its functional companions in skin desquamation, KLK5 and KLK7 [186].

Eventually, KLK15 (S01.081) contains the uncommon Glu189, which was already discussed in Section 3.5 as an element that could shift the P1 specificity from Arg to Lys. However, nearly 1400 reported cleavages in MEROPS corroborate a preference for P1-Arg. Moreover, it features another unique insertion among the KLKs, with additional 10 residues in the 148-loop, which according to AlphaFold forms no distinct secondary structure (Figure 10F).

Surprisingly, the molecular surface appears to be divided in an extended negatively charged region, comprising the S1and S1′ pockets and the base of the 148-loop, with polar S2 to S4 pockets, whereas bordering the prime side a positively charged exosite I can be observed. KLK15 comprises the potential divalent cation ligands Glu70, Asp77, and Glu80, which may represent the stimulatory binding site as 2.5 mM Ca^2+^ enhanced the activity with the substrate Val-Pro-Arg-AMC [114].

## 5. Conclusions

Undoubtedly, KLK3/PSA is among the KLKs which are the major targets for all kinds of academic and commercial pharmaceutical research, including the most advanced novel therapeutic approaches. However, accumulating evidence underscores the broader clinical relevance of nearly all KLKs, either as potential targets in various cancers, e.g., ovarian cancer, or in other diseases [19,20]. In prostate cancer, KLK2 and KLK4 have become valuable diagnostic tools and drug targets, as in the 4K test and with a promising new monoclonal Fab [77,140]. In principle, the ternary complexes of KLK3/PSA with a bound substrate and a stabilizing antibody have already provided the crystal structural blueprint for corresponding approaches [161]. A variety of molecular strategies is currently being pursued to counteract dysregulated KLK activity. Inhibitory antibodies, nucleic acid-based DNA and RNA aptamers, and gene-silencing approaches with siRNA or miRNA have a great potential to directly inhibit dysregulated KLKs or interfere with pathological processes in which they are participating. Probably, aptamers have great potential, since they may be engineered like other biomolecules either with non-natural nucleotides or by introducing special configurations as DNA quadruplexes [293]. In particular, the skin-derived KLK5, KLK7, and KLK14 have recently gained increasing attention as promising therapeutic targets in inflammatory skin disorders and cutaneous malignancies, such as the widespread atopic dermatitis and the rarer Netherton syndrome [294]. Accordingly, a bispecific antibody that binds KLK5 and KLK7 appears to be a further step towards therapies for these skin diseases [75]. Such interventions hold considerable promise for either directly neutralizing aberrant KLKs or modulating the downstream molecular cascades in which these proteases play critical roles. Importantly, the success of these approaches depends on a comprehensive understanding of the structural and mechanistic principles governing KLK function, including their interactions with substrates, cofactors, and inhibitors.

AlphaFold3 and related AI software are great new tools that can help many scientists in the life sciences to efficiently plan their research projects and speed up their experiments. However, according to the terms of service, AlphaFold output cannot be used in any automated system for ligand binding, i.e., in drug design processes. All AlphaFold3 predictions comprise confidence outputs correlating with prediction accuracy, whereby the general limitations are discussed in the latest publication of the developers [281]. Typically, flexible loops have lower confidence levels, even 4.4% of chirality violations, and sometimes overlaps or clashes occur. A list of the AlphaFold server clearly defines which biologically common ligands, ions, and post-translational modifications can be modeled (https://alphafoldserver.com/about, accessed on 30 October 2025). Certainly, AlphaFold, RoseTTAFold, ProteinBERT, and other programs or servers are constantly improved, as well as their integration into the established web services of Swiss-Model, Rosetta, and I-TASSER [295]. Predicted biomolecular structures can even improve experimental structure determination itself, regardless of the methods NMR, cryo-EM, and X-ray crystallography. A simple example may explain this assertion. Flexible surface loops often prevent crystallization even of relatively small biomolecules such as trypsin-like serine proteases. For example, KLK3/PSA had to be stabilized by various antibodies, which bound the 99/kallikrein loop before it yielded diffracting crystals [10,161]. However, Al software still faces problems with predictions for biomolecular complexes, which was recently confirmed by docking studies with such protein models [296].

Furthermore, AI cannot predict cryptic ligand binding sites, which only form in an allosteric manner once a substrate or inhibitor complex is analyzed by structural biological methods [297]. Such cryptic sites with high affinity have been discovered in proteases and may well be exploited for specific interactions with drug compounds. Therefore, high-resolution structural biology remains indispensable for unraveling conformational dynamics, regulatory mechanisms, and for guiding the rational design of selective KLK modulators. Parallel to this, the identification of potent and specific KLK inhibitors remains a major challenge within drug discovery.

In silico mutations as performed for a P1-Phe with reversely bound P1′–P4′ peptide ligands and permutations of all 20 natural canonical amino acids binding to a KLK7 model (Figure 8E), can pave the way for far more efficient computational inhibitor design than screening of huge libraries alone [298]. These targeted simulations enable a more efficient and mechanistically informed design of peptide- and small-molecule inhibitors, offering higher specificity and predictive accuracy than virtual and empirical library screening alone. In addition, this general approach can be applied to target proteins, such as the pheromone binding protein 1 of a moth for key residues and their interaction with several odorants [299]. Probably, this ground-breaking method can be utilized for similar techniques of virtual fragment-based design and become the most powerful tool in strategies of drug design. Altogether, AI-generated models will facilitate further functional studies of KLKs 9 and 11–15, as well as in silico methods for screening of new potent inhibitory lead compounds. Beyond any doubt, these KLKs represent interesting targets in the context of various cancers, but also in case of some conditions such as spinal cord injury, viral infections, and some very rare diseases (Table 1). In addition, structural studies will benefit from reliable predictions by allowing the filtering of more promising targets, e.g., by modifying problematic targets by stabilizing mutations.

Artificial intelligence is increasingly impacting all stages and variations in drug design, starting with protein target and lead compound identification, lead optimization, de novo drug design, or drug repurposing, as well as accelerating preclinical testing and clinical trials [300]. Apparently, structure-based drug design has just begun to utilize the novel capabilities of AI, such as targeting protein structures in deep learning methods with flexible instead of static structures and cofolding of ligands and “receptor” protein [301]. RosettaFold All-Atom employed a transformer architecture with all kinds of molecules and ions, while AlphaFold3 added diffusion-based coordinate generation, which improved the accuracy of interactions and new open-source software like Chai-1134 and Boltz-2 are easier to train. Altogether, AI deep learning methods are significantly accelerating the whole process of target identification, drug discovery, lead optimization, and preclinical and then clinical trials to FDA and EMA approval [302]. Whereas the traditional process took about 12 years on average, the first four steps may now require less than half of the time. Looking further ahead into the future, the field of KLK-directed, in particular and general, drug design may shift from the paradigm of pathology orientation toward a more holistic view with disease-modifying approaches and system therapeutics [303]. Recent progress was made with coevolutionary analysis and deep-learning-based protein structure prediction within the human proteome, resulting in about 18,000 potential protein–protein interactions [304]. Such an integrated perspective will be essential to address the multifaceted physiological and pathological roles of KLKs within proteolytic networks and to fully harness their potential as molecular targets in precision medicine to the benefit of the human patients.

## Figures and Tables

**Table 2:** Physiological functions of KLKs and genetic diseases or gene-related conditions. Semen liquefaction cascade means the proteolytic network for the activation and regulation of KLK2 and KLK3/PSA, which degrade the semenogelins 1 and 2. *Amelogenesis imperfecta* is a genetic disease which causes malformation of enamel resulting in brittle teeth. The suggested physiological functions of KLKs 9, 10, 12, 13, and 15 are very likely, whereas additional evidence is required. Since in the past and occasionally in the current literature alternative names for KLKs are used, a list of the most common ones is provided in Appendix B as Table A1.

KLK	Physiological Function	Non-Cancer Diseases or Disorders
KLK1	blood pressure regulation [89]	Asthma [90], viral infections (SARS-CoV-2) [91]
KLK2	KLK3/PSA activation, semen liquefaction [92]	-
KLK3	semen liquefaction [92]	-
KLK4	semen liquefaction cascade [93], enamel formation [94]	*Amelogenesis imperfecta* [95]
KLK5	skin desquamation [96]	Netherton syndrome [97]
KLK6	regulation of myelin homeostasis in brain [98]	multiple sclerosis [99], Alzheimer’s disease [100] ^1^
KLK7	skin desquamation [96], insulin degradation [101]	Netherton syndrome, atopic dermatitis [97]
KLK8	memory formation [102] ^2^, wound healing [103]	bipolar disorder, impaired memory [104] ^2^
KLK9	immune regulation [105]	spinal cord injury related neurotoxicity [106]
KLK10	immune regulation [105]	Alzheimer’s disease [107]
KLK11	semen liquefaction cascade [93]	Mendelian disorders of cornification [108]
KLK12	control of angiogenesis [109]	influenza virus activation [110]
KLK13	ECM remodeling [111]	Peridontitis [112]
KLK14	skin desquamation [96]	Netherton syndrome [97]
KLK15	Spermatogenesis [113], ECM remodeling [114]	hypermobile Ehlers-Danlos syndrome [115]

^1^ KLK6 is called Neurosin, ^2^ KLK8 is called Neuropsin.

**Table 3:** Most frequent cancer types associated with KLKs according to the *Human Protein Atlas* (https://www.proteinatlas.org/, accessed on 10 December 2025), based on cytoplasmic immunostaining or significant protein expression found by mass spectrometry.

KLK	Cancer Type
KLK1	Prostate cancer, thyroid cancer, glioma
KLKs 2, 3, 4, 11	Prostate cancer
KLK5	Prostate cancer, glioma; thyroid, liver, pancreatic, colorectal, renal and ovarian cancer
KLK6	Lung cancer
KLK7	Skin cancer
KLK8	Skin cancer, breast, cervical and ovarian cancer; head and neck, colorectal and endothelial cancer
KLK9	Skin cancer, head and neck, urothelial and cervical cancer
KLK10	Lung adenocarcinoma
KLK12	Breast cancer [116], otherwise no immunostaining in malignant tissues
KLK13	Prostate cancer; urothelial, cervical, and lung cancer
KLK14	All cancers (according to cytoplasmic tissue staining)
KLK15	Prostate cancer, skin cancer

## Data Availability

No new data were created or analyzed in this study. Data sharing is not applicable to this article.

## Appendix B

**Table A1:** Most common alternative names of KLKs. “Neuropsin” for KLK8 is still used in the current literature referring to brain and neuron physiology.

KLK	hK	Alternative Names for KLKs
KLK1	hK1	glandular/kidney/pancreatic/submandibular/submaxillary/urinary kallikrein
KLK2	hK2	human glandular kallikrein 1 (hGK1)
KLK3	hK3	prostate-specific antigen (PSA), APS, P-30 antigen, seminin, gamma-seminoprotein, semenogelase
KLK4	hK4	prostase, enamel matrix serine proteinase 1 (EMSP1), kallikrein-like 1 protein (KLK-L1), PRSS17
KLK5	hK5	stratum corneum tryptic enzyme (SCTE), kallikrein-like protein 2 (KLK-L2)
KLK6	hK6	neurosin, myelencephalon-specific protease (MSP), BSSP, PRSS9
KLK7	hK7	stratum corneum chymotryptic enzyme (SCCE), PRSS6
KLK8	hK8	neuropsin, ovasin, tumor-associated differentially expressed gene 14 (TADG-14), PRSS19
KLK9	hK9	kallikrein-like protein 3 (KLK-L3)
KLK10	hK10	normal epithelial cell-specific 1 protein (NES1 protein), PRSSL1
KLK11	hK11	hippostasin, TLSP peptidase, PRSS20
KLK12	hK12	kallikrein-like protein 5 (KLK-L5)
KLK13	hK13	kallikrein-like protein 4 (KLK-L4)
KLK14	hK14	kallikrein-like protein 6 (KLK-L6)
KLK15	hK15	prostin, prostinogen, ACO protein
